# Variable and dynamic associations between hot weather, thermal comfort, and individuals’ emotional states during summertime

**DOI:** 10.1186/s40359-024-02005-z

**Published:** 2024-09-27

**Authors:** Kimberly L. Meidenbauer, Kathryn E. Schertz, Peiyuan Li, Ashish Sharma, Tiara R. Freeman, Elizabeth A. Janey, Andrew J. Stier, Anya L. Samtani, Kathryn Gehrke, Marc G. Berman

**Affiliations:** 1 Department of Psychology, Washington State University, P.O. Box 664820, Pullman, WA 99164-4820 USA; 2 Department of Psychology, University of Chicago, Chicago, IL USA; 3 Department of Psychology, University of Michigan, Ann Arbor, MI USA; 4 Discovery Partners Institute, University of Illinois System, Chicago, IL USA; 5 Environmental Science Division, Argonne National Laboratory, Lemont, IL USA; 6 The Santa Fe Institute, Santa Fe, NM USA

**Keywords:** Heat, Affect, Thermal discomfort, Ecological momentary assessment

## Abstract

The effects of heat exposure on negative affect are thought to be central to the observed relationships between hot summer days and deleterious outcomes, such as violent crime or mental health crises. As these relationships are likely to be magnified by the effects of climate change, a better understanding of how consistent or variable the effects of hot weather on affective states is required. The current work combines data gathered from an ecological momentary assessment (EMA) study on individuals’ thermal perceptions, comfort, and affective states in outdoor environments during their daily lives with high spatiotemporal resolution climate-modeled weather variables. Using these data, associations between objective weather variables (temperature, humidity, etc.), perceived heat (thermal perception and comfort), and affective states are examined. Overall, objective weather data reasonably predicted perception and comfort, but only comfort predicted negative affective states. The variance explained across individuals was generally very low in predicting negative affect or comfort, but within-person variance explained was high. In other words, while there may be a relatively consistent relationship between temperature and psychological experience for any given person, there are significant individual differences across people. Age and gender were examined as moderators of these relationships, and while gender had no impact, participant age showed several significant interactions. Specifically, while older adults tended to experience more thermal discomfort and perceived higher temperatures as hotter, the relationship between discomfort and negative affect was lower in older adults. Taken together, these results emphasize the importance of thermal discomfort specifically in predicting negative affect, as well as the high inter-individual variability in thermal perceptions and comfort for the same ambient temperatures.

**Supplementary Information:**

The online version contains supplementary material available at 10.1186/s40359-024-02005-z.

## Introduction

The relationship between heat stress and negative emotions is a well-documented one, and a topic of increasing importance as our planet experiences hotter and more variable conditions due to climate change. The heat-affect link is an essential component of several noted patterns of maladaptive behaviors during summertime, including increases in violent crime and an uptick in suicides and mental health hospitalizations on hot days [1]. For example, key theories of this heat-violence relationship, such as the Temperature-Aggression Hypothesis [2], emphasize that uncomfortable heat leads to negative emotions such as irritability and anger, thereby making it more likely that individuals will react aggressively if provoked. Other work has identified a broad range of negative affective responses due to heat stress, including fatigue, anxiety, frustration, and hostility [3–5], which may be what partially drives the rising of mental health hospitalizations with increasing ambient heat in summertime [6, 7].

Prior work testing these relationships has taken two primary approaches. One approach involves testing these effects by linking time and location-based meteorological data to national surveys [3], hospitalization reports [7], crime incidence [8, 9], or other large-scale aggregated datasets. This approach has high ecological validity, demonstrating the relationship between hot weather and real-world outcomes. However, the correlational nature of these designs and the lack of detailed data on individuals’ emotional states makes it difficult to draw strong causal conclusions about the importance of the heat per se and whether the behaviors are indeed driven by changes in negative emotions. As heat stress may cause other alterations in behavior (i.e., impairments in cognition, changes in activity patterns, and disrupted sleep [10–12]), the centrality of the negative emotional responses in predicting such behaviors is hard to test.

The other key approach, adopted primarily in psychological research, involves experimentally manipulating heat exposure and directly testing its effects on different metrics of negative emotional states. These measures may include self-reported affect, physiological arousal, and/or aggressive behaviors [2, 12–14]. Studies adopting this approach benefit from higher experimental control and a more direct measure of the negative emotional response, but cannot directly speak to how these negative emotional states may impact real-world outcomes or how these results may differ if examined in individuals’ daily lives.

Ideally, these two levels of analysis could be linked to create a more psychology-informed model of the relationship between hot temperatures and key outcomes of interest, such as violent crime or mental health hospitalizations. For example, a long-standing debate within the heat and violence investigation involved the shape of the relationship between these two variables. While some studies found a linear relationship (higher heat, more violent crime) [15–17], others found a curvilinear relationship (both increase to a point, and then the relationship becomes negligible or negative) [18, 19]. It was proposed that the curvilinear relationship may be due to a threshold of negative affect - at a certain point, negative affect is superseded by the fatigue and lethargy that acute heat induces [20]. Some laboratory investigations provided support for this mechanism -- the negative-affect escape model suggests that at a point, aggression drops off as people experience more heat as their goals shift away from anger and aggression towards removing themselves from the situation [14, 20]. Though the majority of this work was conducted several decades ago, and there remains some disagreement on the ‘true’ shape of the relationship, this example demonstrates in principle how the two levels of analysis may be linked in order to better understand a complex pattern.

The ultimate goal of this study was to do precisely this -- generate a better understanding of thresholds of heat stress from temperature and other weather variables which may have a more or less consistent impact on negative emotional states. Subsequently, these results could be used to generate a ‘negative-affect-centric’ model predicting violent crime and mental illness incidence from heat exposure, thereby bridging these two levels of analysis.

We aimed to do this by leveraging data from an ecological momentary assessment (EMA) study design with a large sample of participants who completed surveys while in different outdoor environments during the summer months in Chicago. These surveys included questions regarding their physical environment (including temperature perception and thermal comfort), as well as current affective states. These surveys were time- and geolocation-locked, allowing for outdoor ambient temperature and other weather variables (relative humidity, wind, solar radiation) to be linked to temperature perceptions, thermal comfort, and affect in our participants. Using a recently developed and validated climate modeling approach with higher spatial and temporal resolution than standard weather data [21], a key goal was to examine whether there were temperatures, or combinations of temperature and other weather variables, that consistently led to negative affective states during the Chicago summer. These results could then be used to inform this negative-affect-centric model and be used to predict actual violent crime rates and mental health hospitalizations during this same period (see broad pre-registration for study aims: https://osf.io/ta82w*).*

### The present study

In light of this, the first step required was to test whether climate-modeled estimates of weather data could reliably predict thermal perceptions, thermal comfort, and affective states in a large and demographically diverse sample of individuals. We start by analyzing these relationships across all participants, testing the predictive power of the climate-modeled weather variables for temperature perception (hot or cold), comfort, and negative affect, as well as associations between participant-reported attributes (perception, comfort, and affect). We also tested whether these variables predict reductions in positive affect as a secondary aim. Subsequently, we examined these relationships as moderated by key demographic variables (e.g., age, gender).

The current study, which is exploratory in nature and focuses primarily on prediction rather than identifying the underlying mechanisms linking heat and affect, examines these effects across individuals (fixed-effects estimates from mixed-effects regression models) and within individuals (from the mixed-effects models). By examining these relationships in real outdoor environments during individuals’ daily lives, and linking the responses to high spatio-temporal resolution climate modeling, the results of this study provide unique insights into the consistency and variability of hot weather’s effects on emotional states.

## Methods

The data reported here are taken in part from a broader project (the “Mapping Chicago Project”, OSF repository link: https://osf.io/pjfcd*).* Only study info relevant to the current aims is reported here. A more detailed methods section including all study procedures can be found in the Supplementary Materials.

### Participants

426 participants were enrolled in the study. They were recruited primarily via social media (Facebook ads, Craigslist) for a 2-week study during the summer of 2022. The targeted N was 400 to 500 participants, which was based on budgetary constraints and time, as our goal was to collect data only during the summer months. Eligibility for the study was determined based on whether participants: (1) were 18 years or older, (2) lived in the city of Chicago, and (3) starting in wave 4, were not living in a Chicago community area where we already had many participants, as we aimed to prioritize geographic diversity within the city. Eligible participants were sent an email with instructions on how to enroll via the ExpiWell app. A series of data quality checks (see QA checks in Supplementary Materials for more details) were performed to ensure that participants were providing real data. From the 426 enrolled, 9 participants were flagged as likely providing partially fraudulent data and excluded from subsequent analyses. Additionally, some participants did not complete the study procedures required to link the background survey with the other surveys completed.

In total, 394 participants completed the baseline survey and 364 completed at least 1 outdoor environment survey, which is the primary survey type analyzed in this study (average = 7.24 surveys per person, total outdoor surveys = 2,637). After removing observations that did not pass QA checks or could not be matched to modeled temperature data, 2293 outdoor surveys were included. Participants were distributed across waves, with 20 participants in Wave 1 (5/31 − 6/13), 79 participants in Wave 2 (6/17 − 7/1), 80 participants in Wave 3 (7/5–7/19), 81 participants in Wave 4 (7/25 − 8/8), 53 participants in Wave 5 (8/14 − 2/28), 63 participants in Wave 6 (8/28 − 9/11), and 59 participants in Wave 7 (9/11 − 9/25).

In line with our aim for demographic and geographic diversity, we collected data from participants in 67 out of a possible 77 Chicago community areas. Participants’ ages ranged from 18 to 73 and the mean age was 35.96 years (SD = 12.29), and for gender, we had 274 participants who identified as female, 104 participants who identified as male, and 13 who identified as nonbinary/gender nonconforming. Participants were given the option to select one or more of a list the following racial or ethnic identities and/or fill in an open-ended option. We had 64 participants identify as Asian or Asian American, 105 as Black or African American, 58 as Hispanic or Latino or Chicano, 4 as Native American or Alaska Native, 2 as Native Hawaiian or Pacific Islander, 5 as Middle Eastern or North African, 183 as White or Caucasian, 6 as another racial or ethnic identity not listed, and 2 preferred not to provide this information. Additionally, 29 participants selected more than one ethnic or racial identity from this list.

### Study procedures

All study procedures were approved by the University of Chicago Institutional Review Board. Immediately upon downloading the ExpiWell app and before completing any surveys, participants provided informed consent. Participants were instructed that some study elements were required and others were optional, but participants were paid for all completed surveys regardless of whether they met the requirements.

All participants were asked to complete a required Background (Baseline) survey, completed via Qualtrics, which took approximately 15–20 min and for which they were paid $25. This survey asked about a variety of different individual differences (e.g., personality, trait impulsivity, depression symptoms, etc.) and evaluations of their home and neighborhood environments. Of greatest relevance to the current work, participants provided their gender and year of birth which was used to calculate age. Participants were also asked to complete between 5 and 10 outdoor environment surveys. These surveys could be completed in any outdoor location in Chicago but each survey was to be in a distinct location. Participants were paid $5 per outdoor survey, which took 3–4 min each.

#### Outdoor environment survey

The outdoor survey contained a number of survey questions for individuals to fill out in their immediate outdoor environments. Specifically, participants were instructed “Please fill out the following survey in an outdoor environment in Chicago. Each survey must be in a unique location (at least 5 blocks apart)”. Of primary relevance to the current work, participants were asked to evaluate their perceived temperature, comfort of the temperature, and the D-FAW scale for current affective states [22]. The D-FAW is a 10-item affective state questionnaire that asks participants to rate the extent to which each emotion word applies to them right now. It is composed of five positively valenced (Happy, At ease, Motivated, Calm, Active) and five negatively valenced (Anxious, Annoyed, Tired, Bored, Gloomy) emotions.

### Climate modeled variables

In addition to the in-situ measurements collected from participants, we used the urbanized Weather Research and Forecast model (uWRF, version 4.0 [23, 24]), to provide the weather simulation over the summer of 2022. The simulation can offer an overview of the near-surface weather conditions covering the walking routes and survey areas, including near-surface air temperature, humidity, wind speed, pressure, and solar irradiance, which are the primary factors affecting outdoor thermal comfort. The weather simulation provides hourly spatial-gridded data frames over the city of Chicago, thus they comprehensively reflect the spatial variance and temporal evolution of heat stress based on the heterogeneity of the urban fabric. It provides additional information with the survey and serves as a reference to the city-wide weather conditions.

**Fig. 1:**
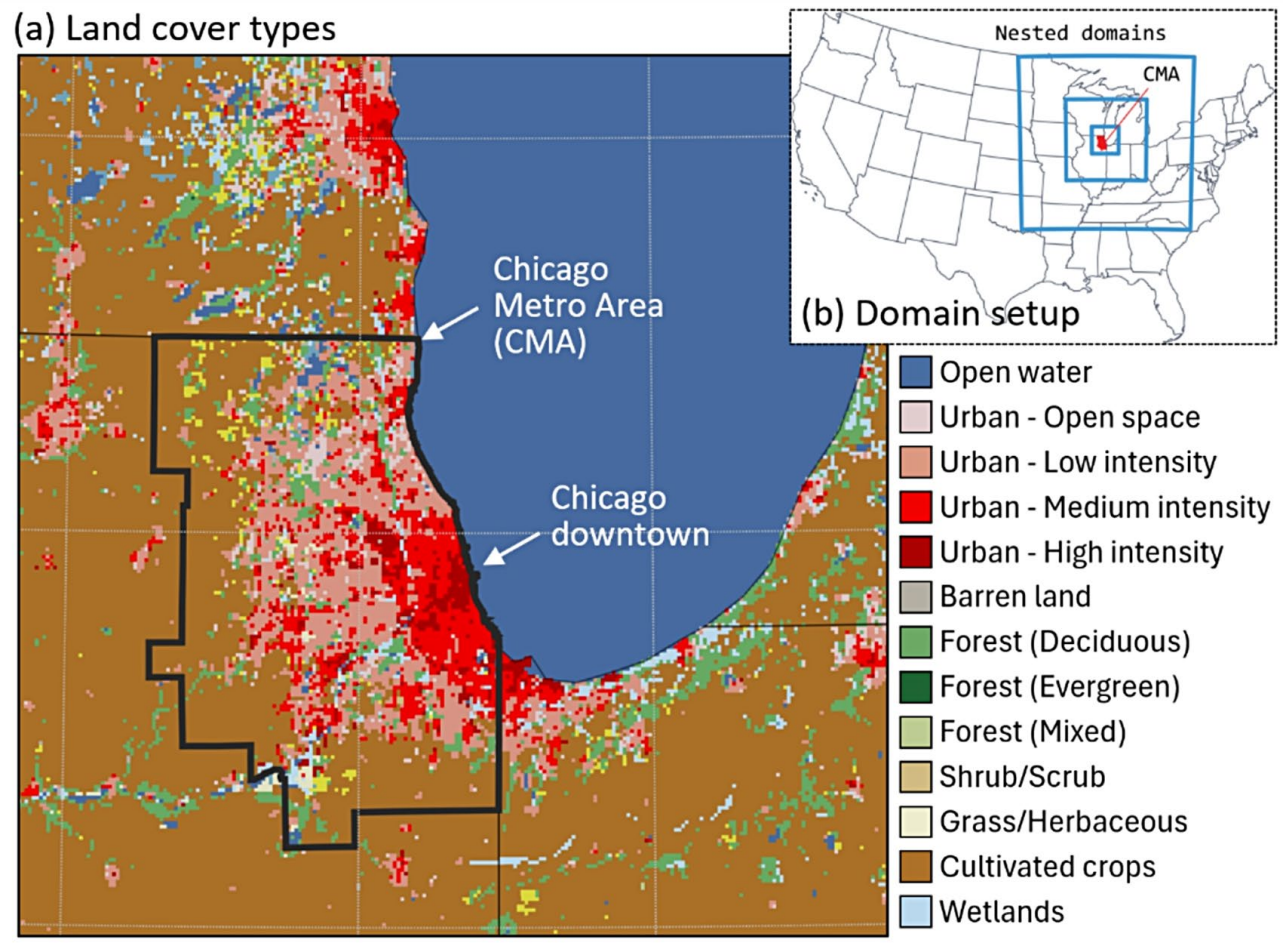
(**a**) Land cover types over the Chicago Metro Area that are used in WRF simulation; (**b**) Location and coverage of nested domain setup used in WRF

The specific configuration of uWRF consists of three two-way nested domains with the outermost boundary covering the east-north central region of the Midwest US and the innermost domain covering the Chicago Metro Area (CMA, Fig. 1a) and its surrounding metropolis (Fig. 1b). The spatial resolutions of the three domains are 9 km, 3 km, and 1 km, respectively. The lateral boundary conditions are from North American Regional Reanalysis (NARR) from the National Center for Environmental Prediction (NCEP, https://rda.ucar.edu/datasets/ds608.0/) with a 32-km horizontal spatial resolution and a 3-hr temporal resolution. In this implementation, we use the single-layer urban canopy model for impervious urban surfaces [23] and the Noah-land surface model (Noah-LSM [25]), for natural land and the previous portion of the urban grids. We also used the WRF Single-Moment 6-class microphysics scheme, which is suitable for high-resolution simulations [26]. Longwave and shortwave radiation are parameterized using the Rapid Radiative Transfer Model (RRTMG [27]). Sub-grid scale cumulus convective parameterization is turned on only for the two outermost domains (9 km and 3 km) corresponding to the Kain-Fritsch scheme [28]. The planetary boundary layer is simulated by the Yonsei University scheme [29], while the surface layer is parameterized by the Monin-Obukhov similarity scheme. The configuration and physical schemes were well-tested in multiple previous studies in Chicago [30, 31]. The simulation period is 2022-05-01 00:00:00 to 2022-09-30 23:00:00 (UTC time, 153 days). Using the time and GPS coordinates of the participants’ outdoor surveys, simulated temperature (degrees C), wind speed, relative humidity, solar radiation, and pressure were linked to each survey.

### Analytic approach

The dataset that was used was collected during the summer months (end of May 2022 through mid-September 2022). Overwhelmingly, the surveys reflected variability between neutral and hot temperatures. However, there were some unseasonably chilly days included in the data as well. As the primary aim is to examine the effects of heat, and as thermal comfort is affected by extremes of either cold or hot (and therefore cannot be modeled linearly), we first removed all surveys in which participants gave the ‘thermal perception’ rating anything less than a ‘4’ (neither hot nor cold), so that the range of responses only included neutral (4) to extremely hot (7). This analysis is the primary one reported in the current paper, and led to the removal of an additional 345 out of 2293 surveys. However, as a robustness check, we ran all analyses using a modeled temperature cutoff of greater than 22 degrees C / 71.6 degrees F (i.e., ‘room temperature’). This led to the removal of 338 out of the 2293 surveys.

Next, histograms of participant-reported variables (positive affect, negative affect, temperature perception, and temperature comfort) were generated to assess the normality of data distributions. Negative affect, in particular, was highly right-skewed, and due to this (and plotted residuals from regression models), models predicting negative affect are conducted on both the raw values (1–7 scale) and a transformed version (using a 1/x function) to generate a less skewed distribution. While the results did not change in most regressions whether negative affect was transformed, QQ-Plots and histograms of the residuals on the 1/x transformed variable were substantially better than those using the raw negative affect variable. Only two statistical tests differed based on the raw vs. transformed variable, and this discrepancy is noted in the results section. For any analyses with multiple predictors, all predictor variables were first z-scored, allowing for direct comparison of the beta values across predictors as needed.

All statistical analyses were conducted using R [32] version 4.1.1. The primary analytical approach used linear mixed-effects regressions predicting the outcome variables from one or more predictors plus a random intercept for each participant (as participants had between 1 and 10 surveys each). As applicable, bivariate relationships were examined via Pearson’s correlations or fixed-effects regressions. For analyses with categorical variables (i.e., gender), independent sample t-tests and fixed-effects regressions were used.

The ‘lmer’ function from the ‘lme4’ package [33] was used for mixed effect regressions. When applicable, the ‘anova’ function from the ‘car’ package [34] was used to compare models. The ‘anova’ function here tests whether the more complex model is significantly better at capturing the data than the simpler model, where significance is defined as a p-value < 0.05 from a χ^2^ test. The ‘summ’ function from the ‘jtools’ package [35] was used to generate the Pseudo-*R*^*2*^ for fixed effects and total (fixed + random) effects. Pseudo-*R*^*2*^ values were calculated using the procedures established by Nagakawa and Schielzeth [36]. Bivariate correlations were conducted using the ‘cor.test’ function of the base R ‘stats’ package and the correlogram was generated using the ‘ggcorrplot’ package [37]. Prediction plots were created using the ‘ggpredict’ function from the ‘ggeffects’ package [38], and scatterplots and descriptive figures were generated using the ‘ggplot2’ [39] and ‘yarrr’ libraries [40]. The function ‘qqPlot’ from the ‘car’ [34] package was used to check model residuals. Full analysis code and output can be accessed on the OSF repository for this project: https://osf.io/4y8eq/.

## Results

**Fig. 2:**
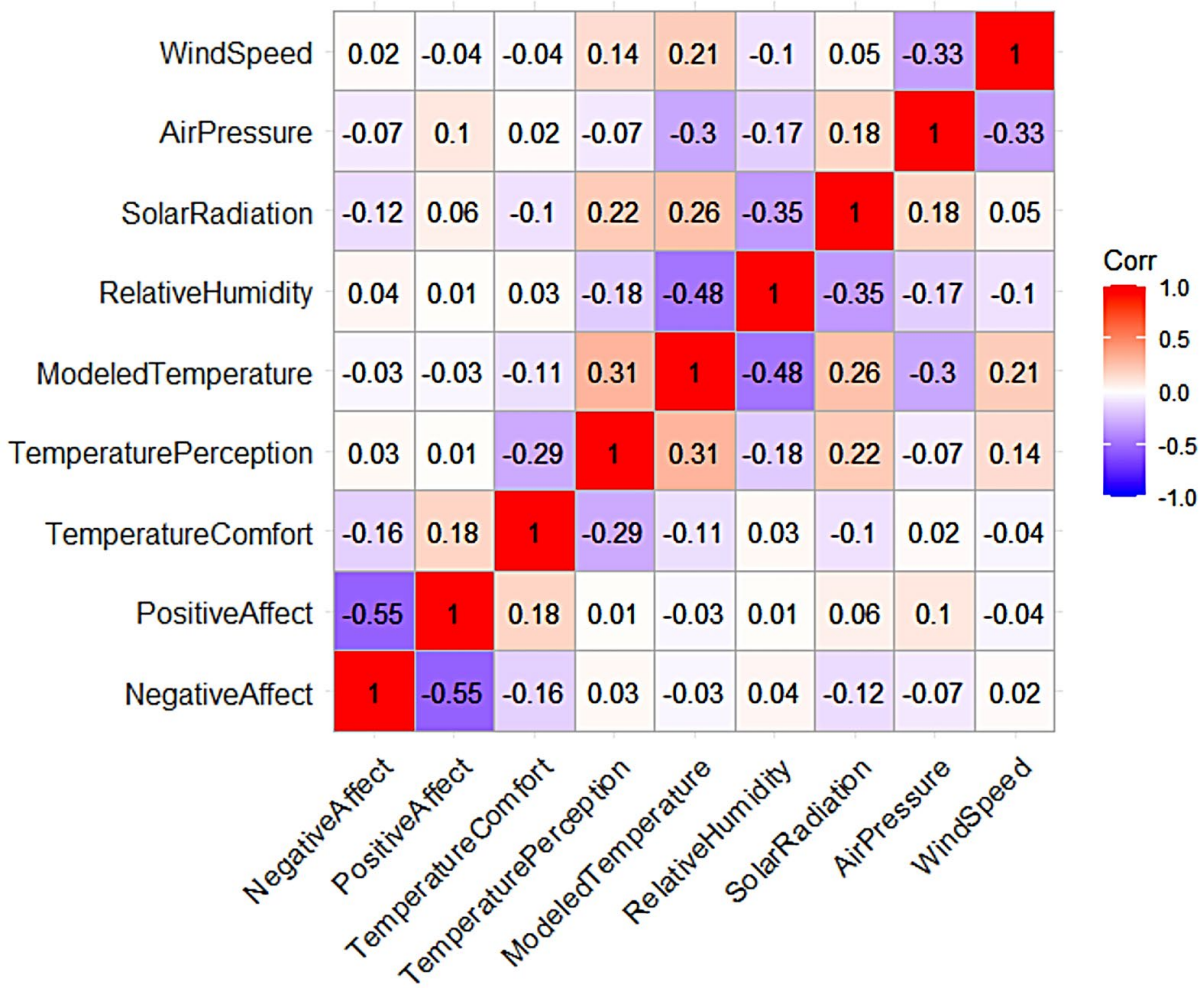
Correlations between variables of interest. Values reflect Pearson’s correlation coefficients

Prior to running regression analyses, bivariate correlations were examined to check for multicollinearity. Figure 2 shows the correlations between the key variables examined in the regressions below. As seen in Fig. 2, none of the key variables of interest show high enough correlation coefficients (i.e., *r* > 0.7) to warrant concerns over multicollinearity.

### Predicting thermal comfort and perception from climate modeled variables

In models predicting both thermal comfort ratings and temperature perception ratings, we ran mixed-effects regressions using model-simulated temperature (variable expected to be the strongest predictor) as well as regressions using temperature, humidity, wind, solar radiation, and air pressure. We then compared the model fits for the simple (temperature only) vs. more complex (all variables) regressions.

Modeled temperature was significantly and positively predictive of thermal perception in the simple model (raw temperature β *=* 0.07, *p* < 0.001). The beta for the unstandardized temperature variable suggests that a 1-degree C increase in temperature is associated with a 0.07 unit increase in temperature perception. In the full model, z-scored temperature (β = 0.25, *p* < 0.001), solar radiation (β = 0.16, *p* < 0.001), and unexpectedly, wind speed (β = 0.06, *p* < 0.001) were all positively predictive of hotter perceptions. In the simple model, the estimated variance explained (pseudo-*R*^*2*^) was 10% for the fixed effects and 25% for the total effect. In the full model, the pseudo-*R*^*2*^s were slightly higher (13% variance explained for fixed effects and 27% for the total), and the model comparison test showed that the model with all weather variables was significantly better (*p* < 0.001), accounting for both parsimony and predictive power. Full model results can be found in Table 1.

**Fig. 3:**
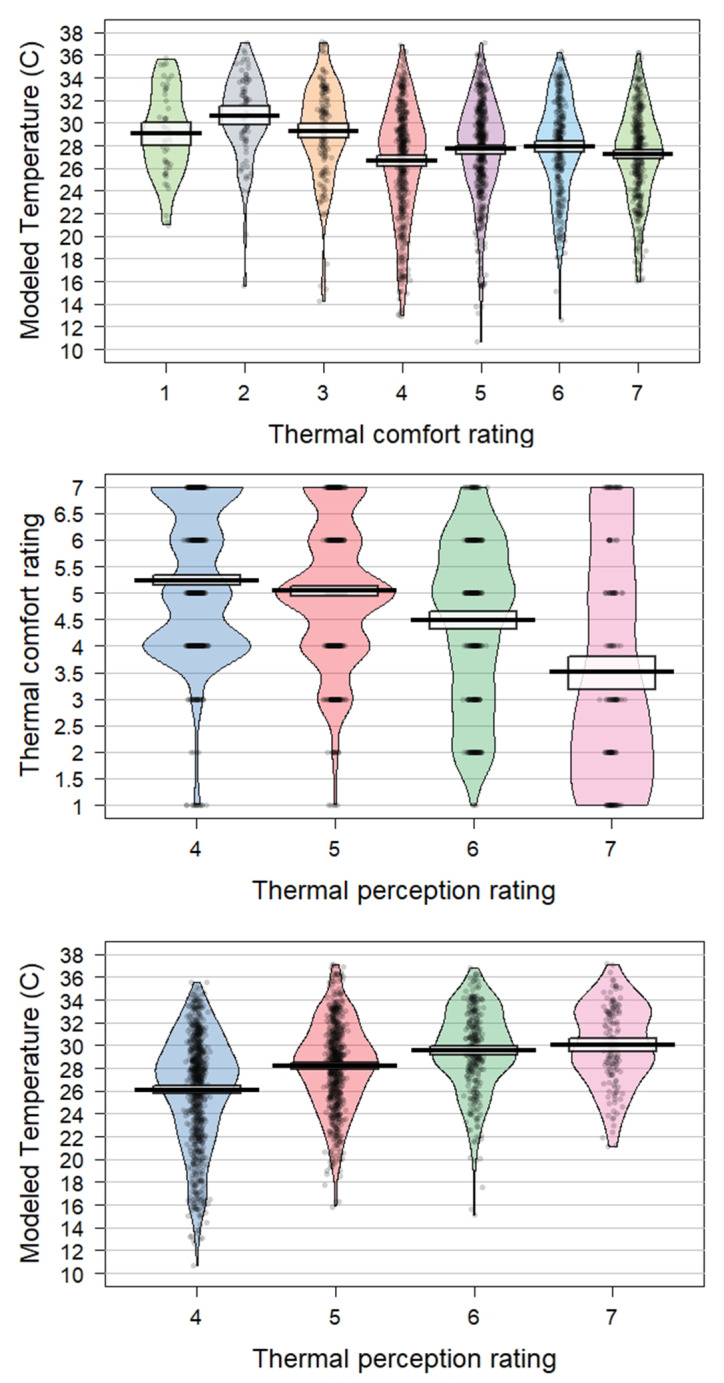
Relationships between modeled temperature, perception, and comfort. Violin plots show distributions of data plotted as a function of Modeled Temperature and Comfort (Top), between Comfort and Perception (Middle), and between Modeled Temperature and Perception (Bottom). Perception ratings are on a 4–7 scale (4 = Neutral and 7 = Very Hot). Comfort ratings are on a 1–7 scale (1 = Very Uncomfortable and 7 = Very Comfortable)

When thermal comfort was the outcome variable, raw modeled temperature (degrees C) was significantly predictive of thermal discomfort in the simple model (β = -0.05, *p* < 0.001). In other words, a 1 degree C increase in temperature is associated with a 0.05 unit decrease in temperature comfort. In the full model, z-scored temperature (β = -0.18, *p* < 0.001) and solar radiation (β = -0.16, *p* < 0.001) were both significantly associated with more thermal discomfort. In the simple model, the variance explained for fixed effects was 2% and 19% for the total effect. In the full model, these increased slightly (3% for fixed effects, 21% for total effect). Results of the model comparison showed that the full model was again better than the simple one (*p* = 0.004). Full model results can be found in Table 2. Relationships between perception, comfort, and actual temperature can be seen in Fig. 3.

**Table 1:** Mixed effects regressions predicting temperature perception. Model includes z-scored simulated weather variables. Estimates are β values with 95% CIs. Fixed effects Pseudo-*R*^*2*^ reports the variance explained across participants, whereas total effects Pseudo-*R*^*2*^ account for fixed effects + participant-level variability

	Model 1	Model 2
Intercept	4.87 ***	4.88 ***
	[4.82, 4.93]	[4.82, 4.93]
Temperature	0.30 ***	0.25 ***
	[0.26, 0.34]	[0.20, 0.31]
Relative Humidity		0.02
		[-0.02, 0.07]
Solar Radiation		0.16 ***
		[0.11, 0.20]
Wind Speed		0.06 **
		[0.02, 0.11]
Air Pressure		-0.00
		[-0.05, 0.05]
N	1948	1948
N (UniqueID)	313	313
AIC	4924.28	4894.70
BIC	4946.58	4939.30
Pseudo-R^2^ (fixed)	0.10	0.13
Pseudo-R^2^ (total)	0.25	0.27

*** *p* < 0.001; ** *p* < 0.01; * *p* < 0.05

**Table 2:** Mixed effects regressions predicting temperature comfort. Model includes z-scored simulated weather variables. Estimates are β values with 95% CIs. Fixed effects Pseudo-*R*^*2*^ reports the variance explained across participants, whereas total effects Pseudo-*R*^*2*^ account for fixed effects + participant-level variability

	Model 1	Model 2
Intercept	4.92 ***	4.92 ***
	[4.83, 5.02]	[4.82, 5.02]
Temperature	-0.20 ***	-0.18 ***
	[-0.27, -0.13]	[-0.27, -0.08]
Relative Humidity		-0.08
		[-0.16, 0.01]
Solar Radiation		-0.16 ***
		[-0.23, -0.08]
Wind Speed		-0.04
		[-0.11, 0.04]
Air Pressure		0.04
		[-0.05, 0.13]
N	1946	1946
N (UniqueID)	313	313
AIC	7026.99	7033.53
BIC	7049.29	7078.12
Pseudo-R^2^ (fixed)	0.02	0.03
Pseudo-R^2^ (total)	0.19	0.21

*** *p* < 0.001; ** *p* < 0.01; * *p* < 0.05

### Predicting affect from perception, comfort, and climate modeled variables

First, negative affect was examined as a function of z-scored thermal comfort and temperature perception only (without the weather variables), and subsequently, modeled by perception, comfort, and simulated weather variables. As before, the two models are compared, and in this case, to test whether the inclusion of ‘objective’ temperature and other weather variables improves the model prediction above and beyond what participants simply report feeling while minimizing model overfitting.

The first model predicted negative affect from z-scored thermal comfort and temperature perception, and only thermal comfort (β = -0.10, *p* < 0.001) predicted negative affect, with no effect of perception (*ps* = 0.86). 1% of the total variance could be explained by the fixed effects and 49% of the variance in negative affect could be explained by the total effect.

When weather variables were included, thermal comfort remained a significant predictor of negative affect, though temperature itself was not predictive. The only weather variable that predicted negative affect was sunshine (e.g., solar radiation), which was associated with less negative affect (β = -0.05, *p* = 0.02). The fixed effects variance explained was only 2%, and the total effect variance explained was 48%. The model comparison did suggest that the more complex model was the better one again (*p* = 0.004). The full model output for these two can be seen in Table 3.

For positive affect, only thermal comfort was predictive across the two models (both β = 0.14, *p* < 0.001), with none of the weather variables or thermal perception as significant predictors. Model comparison suggested that the simpler model was better in this case, as it was more parsimonious and not better than the more complex model in predictive power (*ps* = 0.16). See Supplementary Materials (Table S1) for detailed output.

**Table 3:** Mixed effects regressions predicting negative affect. Model includes z-scored simulated weather variables. Estimates are β values with 95% CIs. Fixed effects Pseudo-*R*^*2*^ reports the variance explained across participants, whereas total effects Pseudo-*R*^*2*^ account for fixed effects + participant-level variability

	Model 1	Model 2
Intercept	2.10 ***	2.10 ***
	[2.03, 2.18]	[2.02, 2.18]
Thermal Comfort	-0.10 ***	-0.10 ***
	[-0.13, -0.06]	[-0.13, -0.06]
Temperature Perception	0.00	0.02
	[-0.03, 0.04]	[-0.02, 0.05]
Temperature		-0.03
		[-0.09, 0.02]
Relative Humidity		-0.00
		[-0.05, 0.04]
Solar Radiation		-0.05 *
		[-0.08, -0.01]
Wind Speed		0.01
		[-0.02, 0.05]
Air Pressure		-0.04
		[-0.09, 0.00]
N	1946	1946
N (UniqueID)	313	313
AIC	4477.49	4500.14
BIC	4505.36	4555.88
Pseudo-R^2^ (fixed)	0.01	0.02
Pseudo-R^2^ (total)	0.49	0.48

*** *p* < 0.001; ** *p* < 0.01; * *p* < 0.05

### Moderation by age

To examine the effects by age, we first conducted mixed effects regressions to examine age as a moderator predicting thermal comfort from temperature and thermal perception from temperature. Age and other predictor variables were z-scored before being entered into the regression model.

**Fig. 4:**
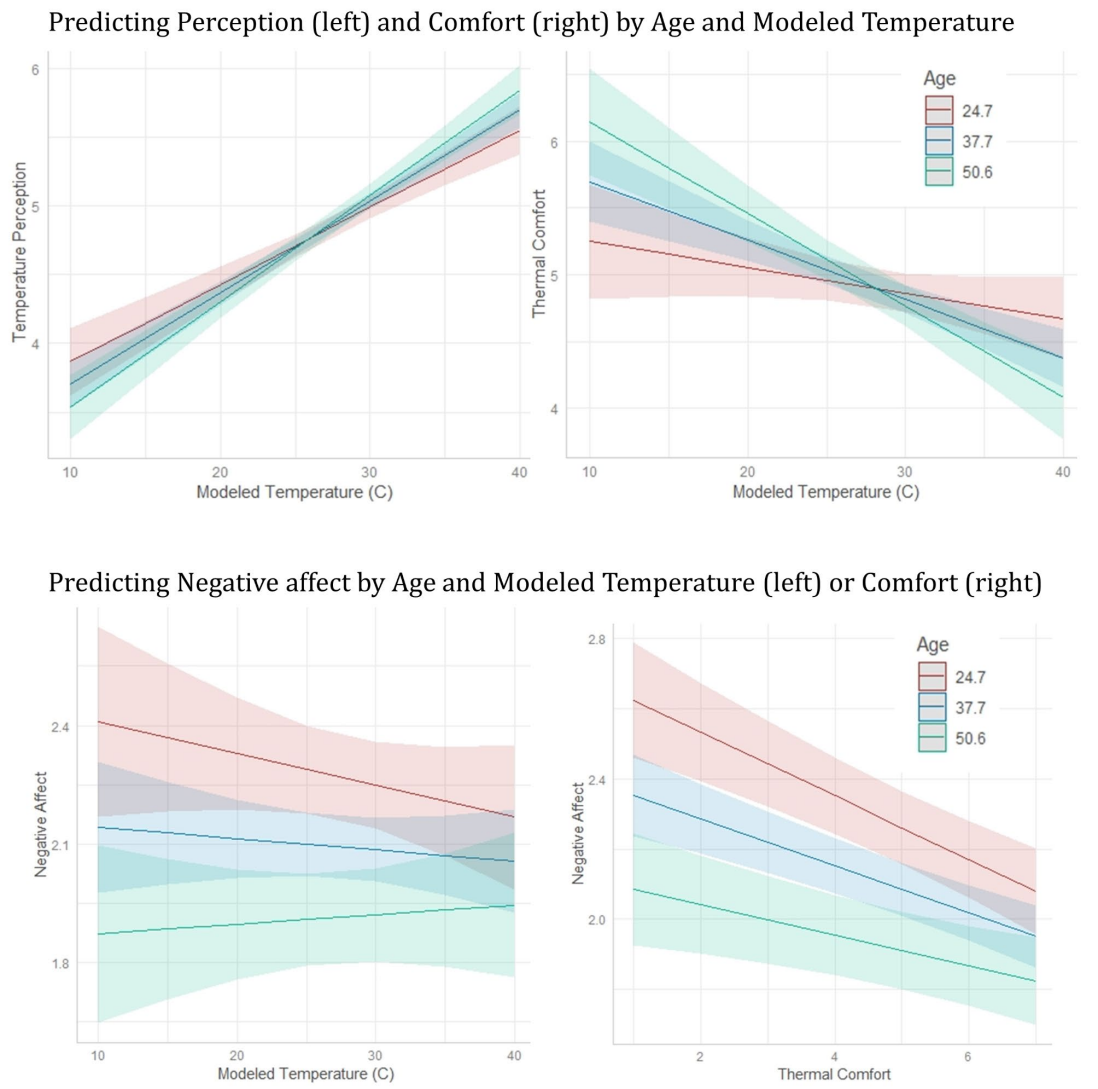
Prediction plots for analyses with age moderator. Prediction plots generated from fixed-effects estimates, split by +/-1 SD from mean age

Results from the regression predicting thermal perception from temperature and age showed a significant main effect of temperature (β = 0.30, *p* < 0.001) and an interaction of temperature by participant age (*p* = 0.02). Plotting the relationship (Fig. 4) showed that the relationship between a hotter perception and higher temperatures was larger in older vs. younger adults. For comfort, the regression showed a main effect of temperature (β = -0.20, *p* < 0.001), but not of age. Again, there was a temperature-by-age interaction (*p* = 0.002). Again, older adults showed a larger relationship between hotter temperatures and more discomfort than younger adults. Full results for the regressions predicting comfort and perception can be found in Table 4.

**Table 4:** Mixed effects regressions predicting thermal comfort and perception from modeled air temperature and age. Model includes z-scored temperature and age. Estimates are β values with 95% CIs. Fixed effects Pseudo-*R*^*2*^ reports the variance explained across participants, whereas total effects Pseudo-*R*^*2*^ account for fixed effects + participant-level variability

	Thermal comfort	Temperature perception
Intercept	4.92 ***	4.88 ***
	[4.82, 5.02]	[4.82, 4.93]
Temperature	-0.20 ***	0.30 ***
	[-0.27, -0.13]	[0.26, 0.34]
Age	0.01	0.02
	[-0.09, 0.11]	[-0.04, 0.07]
Temperature*Age	-0.11 **	0.05 *
	[-0.18, -0.04]	[0.01, 0.09]
N	1946	1948
N (UniqueID)	313	313
AIC	7040.26	4944.12
BIC	7073.71	4977.57
Pseudo-R^2^ (fixed)	0.02	0.11
Pseudo-R^2^ (total)	0.20	0.25

*** *p* < 0.001; ** *p* < 0.01; * *p* < 0.05

Next, we examined whether age moderated any relationships between negative affect and (1) thermal comfort, (2) thermal perception, and (3) actual temperature.

The first model (1) showed a significant interaction between age and thermal comfort in predicting negative affect (β = 0.04, *p* = 0.02). Here, younger adults showed a stronger relationship between thermal comfort and negative affect, whereas the relationship was weaker in older adults (See Fig. 4). Full Model output can be seen in Supplementary Materials Table S2.

The second model (2) predicting negative affect from perception and age did not yield an interaction between perception and age (*ps* = 0.9). Full model output can be found in the Supplementary Materials Tables S3.

Lastly, when examined as a function of (3) actual temperature and age, 1/x transformed negative affect yielded an interaction between temperature and age (β = -0.01, *p* = 0.034), where there was a stronger relationship between temperature and negative affect in younger adults vs. older adults. However, when raw negative affect scores were examined, the interaction between temperature and age was not significant (*ps* = 0.21). Full output for both versions of these models can be found in the Supplementary Materials Table S4 and S5.

A parallel set of analyses were conducted examining positive affect. Overall, older adults showed more positive affect overall (indicated by significant main effects in all three regressions). A significant interaction between age and comfort was found where the relationship between comfort and affect was larger in younger adults. No other significant main effects or interactions were found in the models using modeled temperature or temperature perception as predictors.

**Fig. 5:**
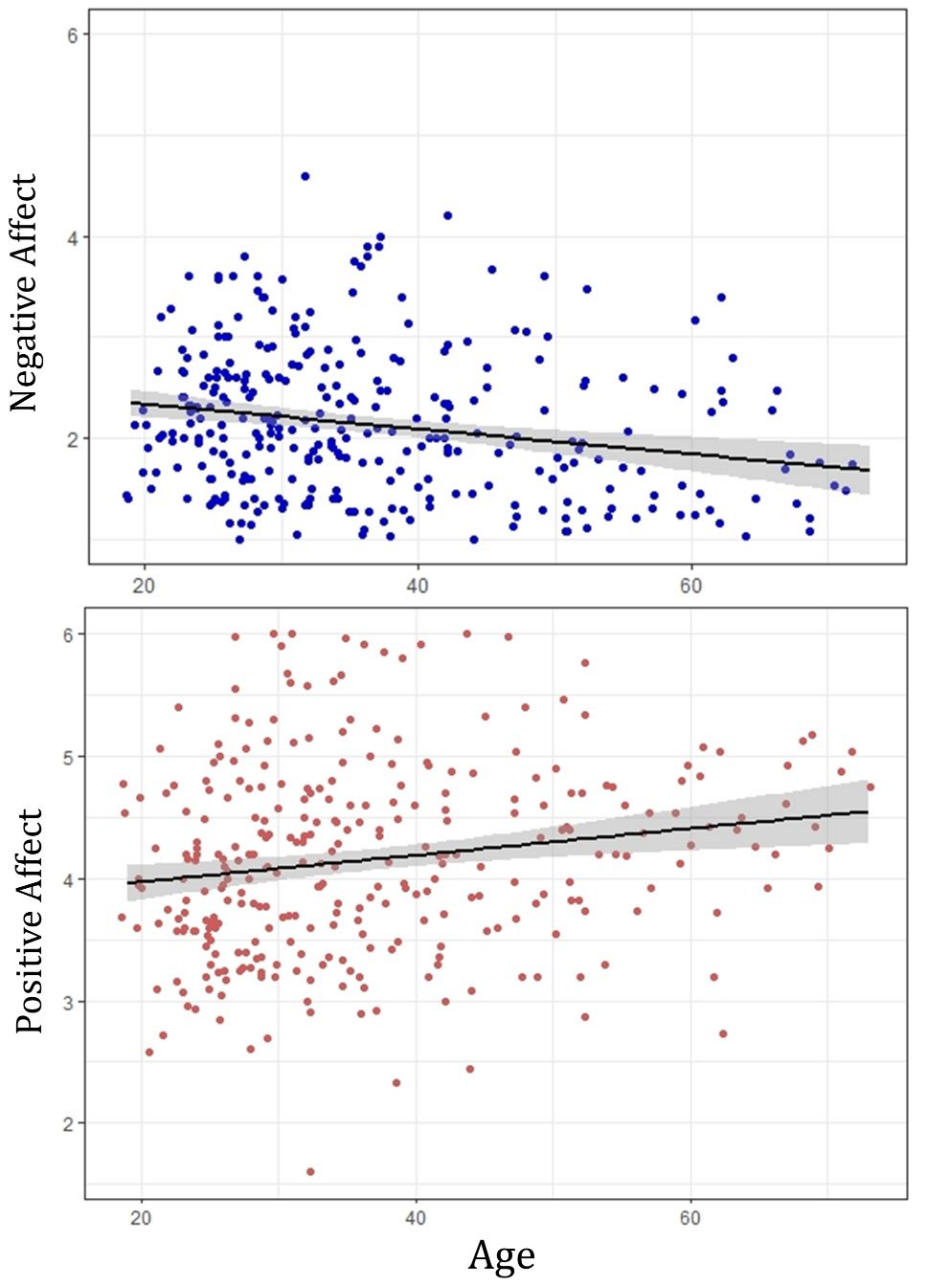
Scatterplots of averaged negative and positive affect by age. Scatterplots with regression line and 95% CI (shaded) for negative affect (top) and positive affect (bottom)

Overall, this pattern of effects suggests that while older adults may be more affected by the actual temperature in terms of how it feels and the discomfort it causes, older adults are generally less likely to experience negative affect and more likely to experience positive affect (see scatterplots in Fig. 5). The overall better affective states in older adults appears important, as while they experience more thermal discomfort, they are less likely to have this discomfort lead to worsened affect compared to younger adults.

### Moderation by gender

In all analyses incorporating gender, we included three categories: female (*n* = 221), male (*n* = 80), and nonbinary or gender nonconforming (*n* = 12). Welch’s independent sample t-tests were conducted to examine overall differences in positive and negative affect between each group irrespective of temperature conditions. While no significant pairwise comparisons were found for negative affect, there were significant differences in positive affect for uncorrected t-tests, wherein male participants had greater positive affect than female participants (*t*(125.4) = 2.66, *p* = 0.009) and nonbinary participants (*t*(15.5) = 2.62, *p* = 0.019), but no differences were found between female and nonbinary participants (*ps* = 0.16). However, the difference with nonbinary participants is not statistically significant if Bonferroni correction for multiple comparisons is applied (α = 0.05/3 = 0.0167). Due to this overall difference between female and male participants, but a lack of difference between female and nonbinary participants, it seemed likely that the reference category for regressions may matter. As such, regressions were run twice: with both the female participants and male participants as reference group.

However, regardless of reference category, no significant effects were found for gender as an independent predictor of temperature perception or comfort (bivariate regressions), as a moderator when simulated temperature was used to predict comfort or perception, or when included as a moderator of the relationship between perception or comfort in predicting positive or negative affect. In sum, while there were gender differences in overall positive affect, no analyses incorporating temperature, perception, or comfort yielded any significant moderation effects.

### Acclimatization effects

Lastly, we examined the effects of Study Wave as acclimatization over the course of the summer months can impact both perception and comfort as individuals become more physiologically adapted to hotter weather. The analyses paralleled the age and gender moderator analyses, where all the models run had an interaction term (“Wave”) and the primary effect of interest was the interaction between Wave and the other variable.

Interactions predicting comfort and perception from study wave and modeled temperature both yielded significant interactions. For comfort, this was a positive interaction (β = 0.07, *p* < 0.001), suggesting that similarly hot temperatures were perceived as more comfortable towards the end of summer. For perception, the interaction was negative (β = -0.02, *p* = 0.02), suggesting that over time, similar temperatures were perceived as cooler. Full output for these analyses can be found in Supplementary Materials Tables S6 and S7. These analyses do suggest that acclimatization is occurring in this sample. However, none of the analyses predicting affective states yielded any significant interactions. In other words, acclimatization did not impact the relationship between affective states and modeled temperature, temperature comfort, or temperature perception.

### Robustness check: exclusion based on modeled temperature

**Fig. 6:**
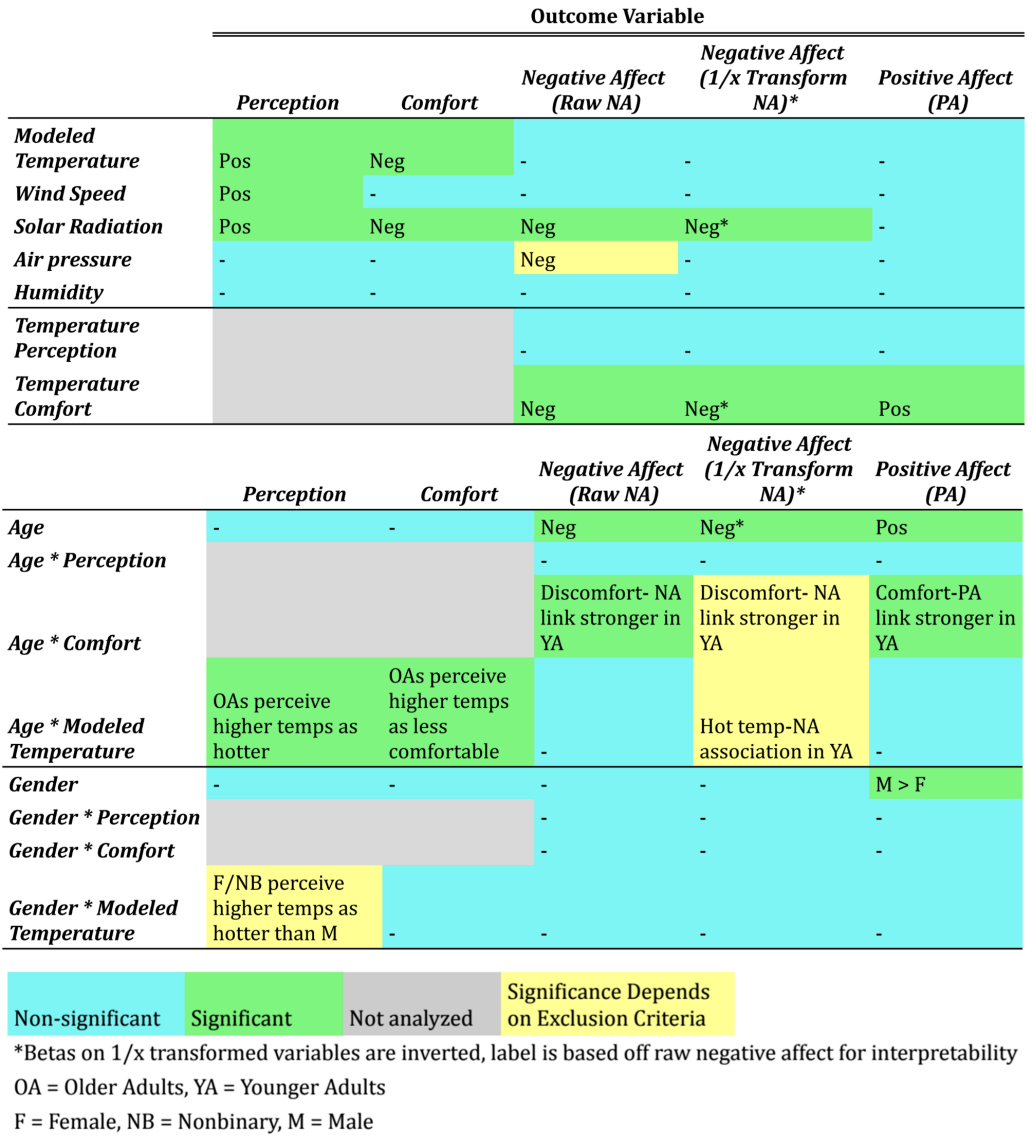
Summary of results: main effects and demographic moderators. Color-coded tables show whether a result is significant (green), non-significant (blue) or significance is affected by how non-hot temperatures are defined for exclusion (yellow). Sign/direction (Positive or Negative) is included for main effects and short description is used for interaction effects

**Fig. 7:**
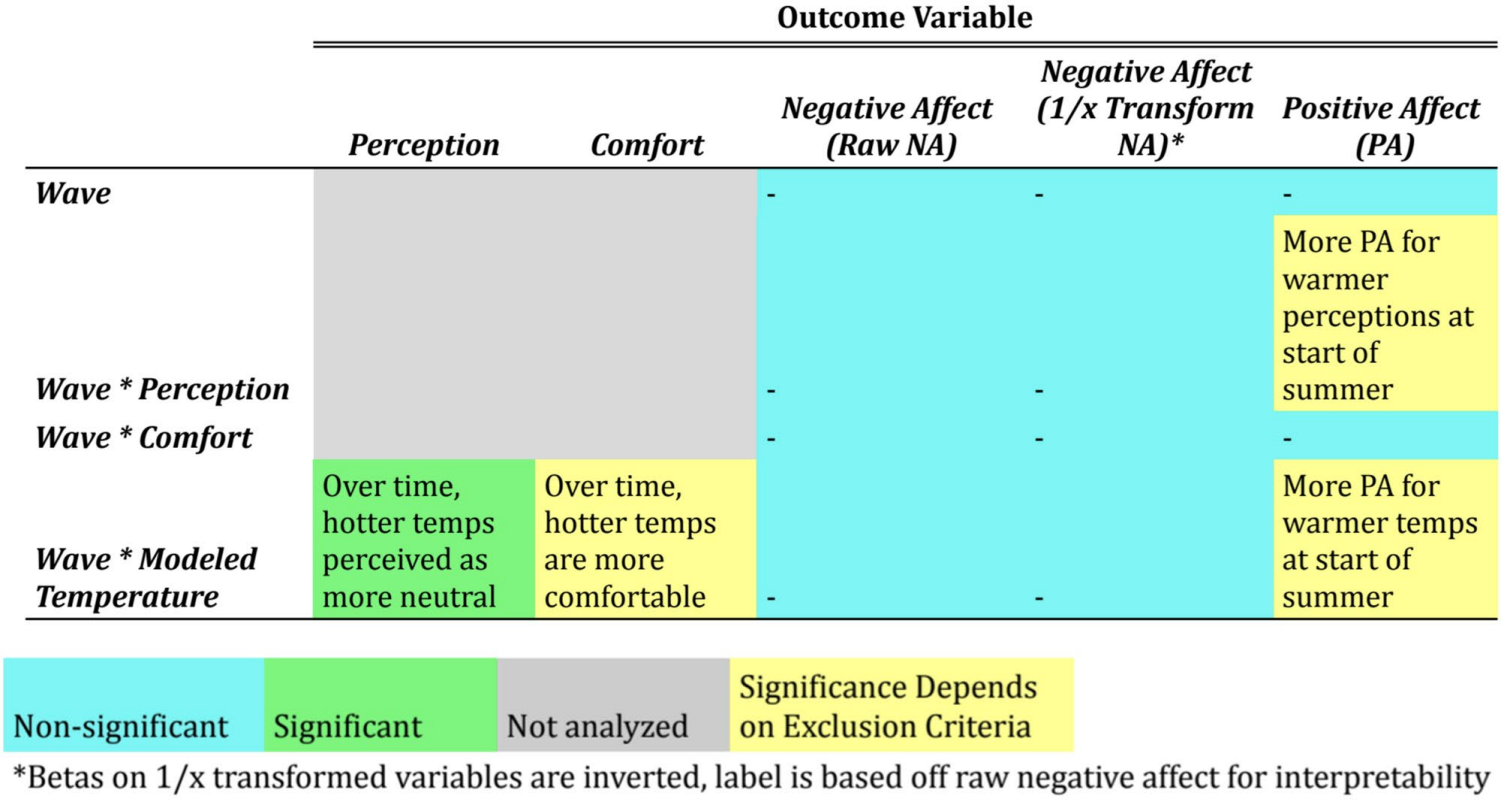
Summary of results: seasonality effects. Color-coded tables show whether a result is significant (green), non-significant (blue) or significance is affected by how non-hot temperatures are defined for exclusion (yellow)

All analyses reported above use data where temperature perception falls between neutral (4) and extremely hot (7). As a robustness check, the same analyses were conducted where modeled temperature (> 22 degrees C) is used as a cutoff rather than perception. The full results of this analysis can be accessed at the OSF project page https://osf.io/4y8eq and can be compared with the identical analysis output with the perception cutoff available on that page. Overwhelmingly, the exclusion approach does not affect the pattern of analyses, though there are a few minor differences with this new threshold. These differences are detailed below, and Figs. 6 and 7 graphically highlight where the exclusion criteria influences the results.

#### Negative affect

In the analyses predicting negative affect from all weather variables, higher air pressure is now a significant predictor of higher raw negative affect (β = -0.05, *p* = 0.041).

#### Age moderator

In the analyses with age as a moderator, the analysis predicting 1/x transformed negative affect from comfort and age was previously significant and is not significant (*p* = 0.06) in the new analysis. In the analyses predicting 1/x transformed negative affect from modeled temperature and age was previously significant but is no longer significant.

#### Gender moderator

While there still proved to be no moderating effect of gender in predicting affective states from any variables examined, this subset of the data did show a weak but significant interaction predicting temperature perception from gender and modeled temperature. Specifically, it appears that the relationship between higher modeled temperature and temperature perceptions is stronger in female (*p* = 0.042) and non-binary/gender non-conforming (*p* = 0.021) participants relative to male participants.

#### Acclimatization effects

The analyses using study wave as an interaction term showed some different results when using this cutoff. The interaction predicting comfort from wave and modeled temperature was no longer significant in this analysis (*ps* = 0.14) suggesting that the effects of acclimatization may be stronger when cooler modeled temperatures are excluded from the dataset. This may be due to the fact that cooler temperatures later in the summer time are perceived as even colder and less comfortable. Full output for these analyses can be found in Supplementary Materials Tables S8 and S9. As with the primary analyses reported, no significant interactions were found with wave in predicting negative affect. However, in this subset of data, there were significant interactions for positive affect. Specifically, hotter temperatures (*p* = 0.02) and hotter perceptions (*p* = 0.01) were associated with more positive affect at the start of summer compared to the end of summer.

## Discussion

A better understanding of the consistency and variability of negative emotional responses to hot weather in summer is needed to generate mechanistically accurate models of the relationships between heat and crime or mental health. One potential caveat to this approach, however, was the possibility that due to inter-individual variability in thermal comfort and perception [41, 42], a psychology-informed model may not emerge from these data. That is, there may or may not be a meaningful threshold of temperature plus other weather variables that consistently lead to both discomfort and negative affect.

The current work sought to test exactly this and identify whether there is indeed sufficient consistency across individuals in terms of their thermal perception, comfort, and emotional responses to hot weather to inform these large-scale models. Through a series of analyses examining the relationships between more objective temperature and weather variables, as well as thermal perceptions, comfort, and affective states, there was limited consistency across individuals, though better predictive power when accounting for within-person effects. Though perception and actual temperature were predictive of thermal comfort, neither predicted negative affect alone. In fact, only thermal discomfort showed a significant association with negative affective states. While a strong test of the underlying mechanisms cannot be performed with the current design, this does suggest that temperature (either via subjective perception or objective modeled weather data) must first be interpreted or appraised as uncomfortable in order for the negative affective response to occur.

Due to this, the current results suggest that using a psychologically-informed model of the heat-crime or heat-mental illness relationship may be a challenge. The variability is such that even at quite high temperatures (38 °C or 100 °F), not all participants perceived the temperature as particularly hot or uncomfortable. Additionally, the fixed effects *R*^*2*^, which reflect the variance explained in negative affect by any heat measure across participants generally (not accounting for within-person effects) were quite low - between 1% and 3%. While the variance explained for the total effect (including within individual effects) was considerably better (pseudo-*R*^*2*^ around 50%), this poses a problem for analyses that focus on temperature and aggregate outcomes where measures of individual experiences are not available. However, it’s worth noting that data of this sort could potentially be used to inform simulation studies in the form of Agent-based Models (ABMs; [43, 44]), which allow for differing levels of heat tolerance and affect across the simulated population. Informed by a mechanistic model of temperature responses, these ABM predictions could then be tested against the actual, observed relationships between heat and crime, and provide insight into the relative contributions of negative affect vs. other factors such as bringing more people outdoors (i.e., what is proposed by the Routine Activities Theory [45]).

These results are consistent with the variability noted in prior research examining individual differences in thermal preferences and comfort, however, these studies have typically utilized relatively small samples and controlled indoor environments, such as office buildings or experimental temperature chambers [46–48]. Studies of this nature also differed in the extent to which they looked at large or small deviations from “room temperature”. It could reasonably be argued that when examined in a relatively restricted range of temperatures, inter-individual differences in comfort may be larger, as people may be more likely to show consensus at temperature extremes than in more temperate conditions. However, the current study suggests that even in a wider range of temperature conditions, the inter-individual differences remain extensive.

In terms of simply predicting perception and comfort from climate-modeled weather data, it is notable that both air temperature and solar radiation were robustly predictive, but other factors such as relative humidity, were not. However, this lack of relationship is likely due to the inverse correlation between temperature and humidity in these data (*r*’= -0.48), and that humidity may increase feelings of heat when it is already hot and individuals are sweating [49], but not when it is more temperate. Solar radiation is also an interesting variable in that it predicts both hotter perception and thermal discomfort, but also better affective states. This is consistent with both the lay notion and empirical evidence backing sunshine as a mood booster [50], and demonstrates the complex dynamics between weather, perception, comfort, and affect again.

Interestingly, the analyses incorporating participant age suggest that the tendency to experience negative affect may indeed be the most important predictor of the heat-affect relationship. Specifically, and consistent with prior research, older adults perceived increasing temperatures as hotter and more uncomfortable than younger adults [51]. However, older adults also showed overall less negative affect and more positive affect. When negative affect was the outcome variable of interest, stronger associations between comfort and objective temperature were found for younger adults. In other words, it seems that though older adults are more physiologically affected by heat (or at least perceive it to be hotter) than younger adults, younger adults’ tendency towards experiencing negative affect heightened the relationship between thermal discomfort and affective states. This effect further emphasizes a relatively weak role of objective or subjective temperature in predicting negative emotions alone and instead demonstrates the importance of individual differences.

This effect is an important addition as studies examining the effects of age and gender have been inconsistent [42]. Specifically, there is evidence that both age and gender may have ‘some’ influence on thermal preferences [47, 49, 52] but the nature of these effects seems varied. For example, a review from 2012 demonstrated that women seem overall more dissatisfied and uncomfortable due to deviations from room temperature in either the hot or cold direction [53]. Additionally, in an indoor setting, older adults appear to perceive the same temperature as slightly colder and less comfortable than younger adults [51].

We failed to find an effect of gender as a moderator in these data. Though men showed slightly more positive affect overall, gender did not yield any interactions in predicting comfort, perception, or affective states. Prior work examining the effects of gender has found mixed evidence- while some studies demonstrate that women prefer warmer temperatures, others find that women are more sensitive to temperature changes in either direction and yet others find no association (see [48, 53] for reviews). The null results in this study suggest a lack of association, however, it is also possible that the unequal sample sizes of men and women reduced the ability to detect an effect here. However, given the reasonably large sample size overall, a more likely scenario is that even if the effect is present, it is a very small one, and may lack practical significance [54]. Additionally, we were very likely underpowered to detect differences between men and women vs. nonbinary/gender nonconforming individuals. This would be an important question to investigate in further research, as much of the prior work has focused solely on a male/female distinction in cisgender individuals without investigating the effects in other gender identity groups.

Acclimatization is another key factor that may influence thermal comfort due to physiological changes which impact thermoregulation efficiency [52, 55]. While this study did not track the same participants over the entire summer, some of our results are consistent with acclimatization effects. Specifically, comfort and perception in hotter temperatures are perceived as more comfortable and neutral at the end of summer vs. the start of summer. However, the time at which participants were tested did not have any impact on negative affective states - the main effect of comfort was still the only predictor of negative affect. This suggests that while acclimatization may impact the relationship between temperature and comfort, discomfort alone predicts negative emotional states regardless of when individuals participated.

There are some limitations to the current work. While we were able to examine these relationships in a highly ecologically valid setting, there are trade-offs to this approach. For one, we cannot account for all variables that may have affected temperature perceptions, including participants’ attire and whether they were in a shaded or unshaded area. Both clothing choice and shade likely influence perception and comfort, increasing the variability in our study relative to a controlled environment where these could be accounted for. However, in the context of the overarching goal of this study (informing large-scale models of the relationship between temperature and affect in predicting aggregate outcomes), we would not be able to account for these possibilities anyway. Thus, while this limits our overall predictive power, it does not affect the predictive power that would be implemented in using a psychologically-informed model of heat and crime or mental illness.

Another important limitation is that the relationship between thermal discomfort and affective states may have been influenced by the act of completing the survey itself. That is, it is possible that reflecting on one’s current feelings of comfort or discomfort may amplify the subsequent reporting of emotional states. However, this may be an important mechanism for understanding the heat-affect relationship, rather than a confound per se. An alternative explanation is that already being in a negative affective state increases the likelihood of perceiving temperature as uncomfortable. This idea is somewhat supported by the age effects - where younger individuals show a stronger relationship between temperature, comfort, and affect than older adults and had an overall stronger tendency towards negative affect. Unfortunately, the current study design cannot disentangle these relationships, and it is very plausible that both mechanisms are at play. Future work in an experimental setting where heat is induced may shed greater light on the underlying mechanisms.

Additionally, while our study provides unique insights into the relationship between real, modeled temperatures as opposed to purely relying on perception or using low-spatial resolution weather records, there are limitations to the WRF model used here. Simulating street-level temperature variability, particularly within the highly heterogeneous urban environment, remains challenging in urban climate studies. While our study synchronized GPS coordinates and time for a matched survey-model comparison, the inherent limitations of current urban climate models prevent us from achieving meter-level resolution across entire cities for extended periods due to impractical computational costs and model instability issues. The 1 km resolution used in this study, although state-of-the-art, may not fully capture the temperature variability experienced by pedestrians. From this perspective, our findings reflect the broader issue of the discrepancy between perceived heat and modeling results. These limitations underscore the need for continued development in both modeling techniques and computational capabilities.

Lastly, while the current work did include an examination of some participant-level factors (age, gender) which may influence the relationships of interest, there are other factors related to vulnerability to heat exposure which we do not account for here. For example, typical heat exposure at home, limited access to AC, low socioeconomic status, and present or past mental health conditions could all exacerbate the effects of heat exposure on emotional states. While outside the scope of the current paper, these are undoubtedly important moderating factors that can be investigated in subsequent analyses.

Despite these limitations, the results of this work provide unique insights into the variability of thermal perceptions, comfort, and affect in a large and diverse sample of individuals in a range of outdoor environments in summertime. By combining not only subjective reports of comfort, perception, and affect, but also high spatiotemporal resolution simulated weather data, the role of ‘objective’ and ‘subjective’ heat was investigated in individuals’ daily lives. As the effects of climate change continue to wreak havoc on weather patterns, a better understanding of the emotional consequences of temperature variability is of crucial importance. This study identifies the importance of individual differences broadly, as well as the specific effects (or lack thereof) of age and gender in our sample. It also paves the way for future work investigating the fundamental mechanisms driving heat-related negative affect in more controlled environments.

## Electronic supplementary material

Below is the link to the electronic supplementary material.

Supplementary Material 1

## Data Availability

Data is available at: https://osf.io/4y8eq/.
