# Supplementary material for: Variable and dynamic associations between hot weather, thermal comfort, and individuals’ emotional states during summertime
Source: BMC Psychol. 2024 Sep 27;12:504. doi: 10.1186/s40359-024-02005-z (PMC11438364; doi:10.1186/s40359-024-02005-z)
Supplement: Supplementary file 1 — Supplementary Material 1 [file 40359_2024_2005_MOESM1_ESM.docx]

# **SUPPLEMENTARY METHODS**

*Full Methods for Mapping Chicago Project*

**Recruitment Method**

Participants were recruited via social media and listservs for seven 2-week study windows between May 31 and September 25. Our targeted N was between 400 and 500 participants based on budgetary constraints and the aim to collect data only during summer months. Specifically, geo-targeted Facebook advertisements were used to recruit participants from the Chicago area, the study was advertised on Reddit.com on several Chicago-specific subreddits, and on Craigslist. An email was also shared with several Chicago-based community organizations to reach a larger sample of Chicagoans. Participants were instructed to email us if interested, and subsequently were given additional information and a pre-screening questionnaire to evaluate eligibility. Participants were eligible to participate in the study if they met the following criteria:

1. Were 18 years or older
2. Lived in the Chicago city proper (zip code provided started with 606##)
3. Were not living in a Chicago Community Area where we already had a large number of participants (applicable starting in wave 4)

Participants who met these criteria were sent instructions on how to enroll in a given study wave using the ExpiWell app. Not all participants who were sent enrollment details downloaded the ExpiWell app and completed the consent form or study procedures. Only those who completed the consent form and who provided non-fraudulent data (see QA section below) on at least one of the surveys are included below.

**Participant info**

A total of 426 participants participated in the study procedures. Of these 426 participants, 9 participants were flagged as potentially providing partially fraudulent data. Participants were from 67 out of 77 Chicago community areas. The mean age was 35.96 years (SD = 12.29, Min = 18, Max = 73). One hundred four identified as male, 274 identified as female, and 13 identified as nonbinary or gender nonconforming. Sixty-four participants identified as Asian/ Asian American, 105 as Black/ African American, 58 as Hispanic/ Latino /Chicano, 4 as Native American/Alaska Native, 2 as Native Hawaiian/ Pacific Islander, 5 as Middle Eastern/ North African, 183 as White/ Caucasian, 6 as another racial or ethnic identity, and 2 preferred not to provide this information. Of the participants who responded to this question, 29 selected more than one ethnic or racial identity from this list. Some participants did not complete the study procedures required to link the background survey or RC-RAGE task with the other surveys completed.

There were 20 participants in Wave 1, 79 participants in Wave 2, 80 participants in Wave 3, 81 participants in Wave 4, 53 participants in Wave 5, 63 participants in Wave 6, and 59 participants in Wave 7.

394 participants completed the baseline survey, 200 participants completed the RC-RAGE task, 364 completed at least 1 outdoor environment survey (average = 7.24 surveys per person, total outdoor surveys = 2,637) and 303 completed at least 1 park evaluation survey (average = 4.08 surveys per person, total park surveys = 1,235).

**Study Procedures**

All study procedures were approved by the University of Chicago Institutional Review Board. Participants were instructed that some study elements were required and others were optional. However, participants were paid based on the surveys they completed regardless of whether they did all of the required surveys.

Upon downloading the ExpiWell app, participants first provided informed consent. Though the study periods were each 2-weeks long, participants were able to download the app, provide informed consent, and enroll in the study up to 3 days before this two week period. All participants were asked to complete a Background (Baseline) survey which took approximately 15-20 minutes and for which they were paid $25. The Background survey was listed as a “required” study component and was completed via Qualtrics and could be done as soon as they enrolled in the study (i.e., before the 2-week window started). Participants were also able to complete an optional “online game” as soon as they enrolled, which was an aggressive tendencies task (the Computer Opponent Version of the RC-RAGE; [(Meidenbauer et al., 2021)](https://paperpile.com/c/D6CCNS/lLJN). The RC-RAGE was embedded in a Qualtrics survey and they were paid $10 for completing the task.

Once the 2-week period started, participants were asked to complete between 3 (minimum) and 5 (maximum) park surveys while in Chicago parks. These surveys were completed in the ExpiWell app. GPS was required to verify that participants were indeed in the location they specified (see QA checks section below for more details). Participants were paid $10 per park survey, which took approximately 5 minutes each. In this time, they were also asked to complete between 5 (minimum) and 10 (maximum) outdoor environment surveys. These surveys could be completed in any outdoor location in Chicago but were required to be 10 distinct locations. Participants were paid $5 per survey for each of the outdoor surveys, which took 3-4 minutes each.

To increase participation throughout the study window, participants were sent reminder messages via the Expiwell app mid-way through the 2-week period. These reminders included information on how many and which of the required surveys participants still needed to be completed and how many days they still had to complete these activities. After data quality checks were completed, participants were paid up to $135 in Amazon gift cards based on how many (valid) surveys were completed.

Background (Baseline) Survey

The background survey included general demographic and geographic questions (year of birth, gender, racial/ethnic identity, educational attainment, income, zip code, community area). Participants also completed a series of questions that assess economic disadvantage (ability to afford food and other essentials such as rent, power, healthcare, etc.), migrant status and experiences (if applicable), and perceptions of their neighborhood. Perceived neighborhood quality and belonging and was evaluated using a subset of questions from the CHART project ([Chicago Health and Activity in Real‐Time (CHART) | NORC.org](https://www.norc.org/Research/Projects/Pages/chicago-health-and-activity-in-real%E2%80%90time.aspx)) and from the MIDUS project [(Brim et al., 1999)](https://paperpile.com/c/D6CCNS/RK2Q).

Participants were also asked about their typical use (if applicable) of Chicago parks. They were asked “How often do you visit parks in Chicago?”, “What kind of activities do you normally go to parks for?” and “What facilities do you care about when you visit parks?”. They were also asked about exposure to excessive heat, physical symptoms experienced from heat, and heat adaptation strategies in a series of questions that were adapted from [(Hayden et al., 2011)](https://paperpile.com/c/D6CCNS/lYfn).

A series of standardized questionnaires were included to evaluate:depressive and anxiety symptoms via the Patient Health Questionnaire-9 (PHQ-9) [(Kroenke & Spitzer, 2002)](https://paperpile.com/c/D6CCNS/i2JF), trait impulsivity via the Barratt Impulsiveness Scale-11 (BIS-11) [(Patton et al., 1995)](https://paperpile.com/c/D6CCNS/2F46), trait aggression via the Buss-Perry Aggression Questionnaire (BPAQ) [(Buss & Perry, 1992)](https://paperpile.com/c/D6CCNS/R9KD), environmental sensitivity via the Highly Sensitive Persons Scale (HSP) [(Aron & Aron, 1997)](https://paperpile.com/c/D6CCNS/KMdS), sense of belongingness via the General Belongingness Scale [(Malone et al., 2012)](https://paperpile.com/c/D6CCNS/klem), life satisfaction via the Satisfaction with Life Scale [(Kobau et al., 2010)](https://paperpile.com/c/D6CCNS/sbg6), Big Five personality traits via the Big Five Inventory-2 Short Form (BFI-2S) [(Soto & John, 2017)](https://paperpile.com/c/D6CCNS/jnXL), self-nature overlap via the Inclusion of Nature in Self scale (INS) [(Schultz, 2002)](https://paperpile.com/c/D6CCNS/m3CP), tendencies towards rumination vs. reflection via the the Rumination and Reflection Questionnaire (RRQ) [(Trapnell & Campbell, 1999)](https://paperpile.com/c/D6CCNS/uT13). Participants also completed the Kirby Delayed Discounting Task [(Kirby et al., 1999)](https://paperpile.com/c/D6CCNS/wozW).

RC-RAGE Task

The Retaliate or Carry-on: Reactive AGression Experiment (RC-RAGE) Computer Opponent Version is an online task which measures costly reactive aggressive tendencies [(Meidenbauer et al., 2021)](https://paperpile.com/c/D6CCNS/lLJN). In the task, participants are instructed to do a tedious “apple gathering” task by clicking around the screen to earn money. Once participants have clicked on 10 apples, 10 cents is added to their bonus. Occasionally, a computer opponent, known as the “robber” appears and steals 5 cents from their bonus. Participants are given the opportunity to retaliate against the robber by shooting and killing him to get 3 cents back. However, doing so resets their progress towards gathering 10 apples. Due to this, it is financially best to ignore the robber if they have only clicked on more than 2 out of 10 apples. Participants are informed of this strategy explicitly during the instructions. Validation studies of this task found that participants who had higher trait aggression and impulsivity, and were in a more hostile emotional state retaliated more when it was financially costly.

Outdoor Environment Survey

The outdoor survey contained a number of survey questions for individuals to fill out in their immediate outdoor environments. Specifically, participants were instructed “Please fill out the following survey in an outdoor environment in Chicago. Each survey must be in a unique location (at least 5 blocks apart). If you are in a park, please take the park survey only. This survey should take approximately 3-4 minutes.”

Participants were asked to evaluate the perceived air cleanliness, air smell, temperature, comfort of the temperature, safety, noisiness, (dis)orderliness, preference (like/dislike) for the place, and whether this is a place they’ve visited before. They were also asked about the number of people present within 1-2 blocks, and to evaluate the social interactions of the individuals around them (i.e., are there people getting along, showing signs of conflict, neutral interactions). To evaluate accessibility, participants were asked whether the sidewalks were well maintained, and whether there was a place to sit in the shade if needed. Additionally, participants were asked to state how connected they felt to their physical and social surroundings, filled out the D-FAW scale for state affect [(Russell & Daniels, 2018)](https://paperpile.com/c/D6CCNS/8LTQ), and indicated the extent to which they thought their physical environment was affecting them [(Schertz, Bowman, Kotabe, Layden, Zhen, Lakhtakia, Lyu, Paraschos, Van Hedger, Vohs, et al., 2022)](https://paperpile.com/c/D6CCNS/YZkL).

Park Survey

The park evaluation survey contained several of the same questions as in the outdoor survey, with some additional park-specific questions. Participants were instructed “Please fill out the following survey while in a Chicago park. Each survey must be completed in a different park. Note that we will use GPS to verify your location inside a park to avoid fake responses. This survey should take approximately 5 minutes.”

After providing the name of the park, participants rated it on perceived cleanliness, naturalness, maintenance, safety (now), safety (at night), noisiness, cleanliness of the air, and their preference (like/dislike) for the park. They indicated whether they had visited the park before, and if so, what facilities they normally use, what activities they normally do, what activities they did at their last visit, and what was their favorite activity to do in the park. They were also asked whether they would visit again, why they would or would not, whether they came to the park to fill out the survey or whether they were coming anyway, and now that they were there, would they stay a while. They were also asked whether they felt they belonged in the park or not. Restorative properties of the park were evaluated using a 4-item version of the Perceived Restoration Scale (PRS) [(Berto, 2005)](https://paperpile.com/c/D6CCNS/iOA3). To assess participants' perceived restoration, they were asked how mentally fatigued and mentally refreshed they felt right now.

As in the outdoor survey, they were asked to approximate the number of people in the park and whether different types of social interactions were present. They were also asked to evaluate the demographic breakdown of others in the park (by gender and age). They also rated how connected they felt to others in their environment, their physical surroundings [(Schertz, Bowman, Kotabe, Layden, Zhen, Lakhtakia, Lyu, Paraschos, Van Hedger, Vohs, et al., 2022)](https://paperpile.com/c/D6CCNS/YZkL), and completed the D-FAW state affect questionnaire [(Russell & Daniels, 2018)](https://paperpile.com/c/D6CCNS/8LTQ). In addition to the adjectives used in the D-FAW (Happy, At ease, Anxious, Annoyed, Motivated, Calm, Tired, Bored, Gloomy, Active), participants rated how Impulsive, Thoughtful, Relaxed, Energized, Irritable, and Compassionate they felt. These adjectives were included based on several theories of how greenspaces may reduce impulsivity (“Impulsive”) [(Schertz, Bowman, Kotabe, Layden, Zhen, Lakhtakia, Lyu, Paraschos, Van Hedger, Rim, et al., 2022)](https://paperpile.com/c/D6CCNS/xny3), increase reflection (“Thoughtful”), reduce stress and induce relaxation (“Relaxed”) [(Ulrich et al., 1991)](https://paperpile.com/c/D6CCNS/niSl), increase subjective vitality (“Energized”) [(Ryan et al., 2010)](https://paperpile.com/c/D6CCNS/PbjO), and improve social interactions, potentially by decreasing anger (“Irritable”) and increasing prosocial orientations (“Compassionate”)[(Passmore & Holder, 2017)](https://paperpile.com/c/D6CCNS/wqzx).

Additionally, biodiversity of the park was evaluated using a short version of the scale developed by [(Fuller et al., 2007)](https://paperpile.com/c/D6CCNS/5K7Q). Lastly, participants were asked to take a photo of the park at the end of the survey. This was used to check that participants were indeed in the park (see QA below) and, as many parks are large and have distinct areas, to determine what participants were seeing as they filled out the survey.

**Data QA Checks & Exclusion Criteria**

A series of data quality checks were performed at the end of the study period to ensure the validity of the surveys:

Outdoor Environment Surveys

As participants were asked to complete surveys in different outdoor locations, an R script which detected very spatially close GPS coordinates off of the survey data was run as well as whether participants were outside of the city of Chicago. This script output which participants and which surveys were potentially in overlapping areas. The coordinates were then put into google maps to manually check whether the latitude/longitude data were indeed within Chicago and/or sufficiently far apart. The results of this manual examination were then logged.

Park Surveys

For the surveys where participants were asked to rate different parks in Chicago, a similar R script was run to flag GPS coordinates that were not within/close to any Chicago parks as specified by the park boundaries shapefile from Chicago’s open data portal. If parks were flagged, the lat/long were again entered into Google Maps in order to determine proximity to the park. If the GPS coordinates did not seem to reflect the park, the specific park survey data were examined to assess authenticity. This was done by examining the photo of the park participants were asked to upload at the end of the survey (making sure it was indeed a picture of that park that seemed real and not imported from online, showed the park in the correct season, and didn't show up on a cursory google search of that park). Additionally, in the case where the picture was not helpful or missing, participants' answers to what facilities and attributes of the park they liked were cross checked to see if they overlapped with actual facilities listed on the Chicago Park District website.

Background (Baseline) Survey

In the baseline survey, we checked for duplicate IP addresses to ensure that we did not have the same individuals participating multiple times and we checked to see if the IP addresses were outside of Chicago. If duplicate IP addresses or outside of Chicago IP addresses were found, other data were examined to check the validity of the participants, such as whether the community area and zip code provided were real areas and/or made sense (i.e. zip and community area should be related). We also checked whether the data participants provided during recruitment matched what they provided in the survey.

Exclusion and Flagging Criteria

Some participants provided clearly fraudulent data, which was identified by several potential mechanisms: the location of one or more surveys indicated they could not have been in Chicago during the time of the study, some participants shared the same IP address as another “participant” and the identity of the participant could not be verified via recruitment (suggesting the same person filled out the survey under a different name), and some participants enrolled in the study without being sent enrollment information (again indicating it was the same person using multiple emails). Per our IRB and consent form, when fraud was detected participants would not be paid. As such, clearly fake participants were not paid and their data from these participants was discarded. These participants were not counted towards the total sample of 426 participants.

However, some participants’ data had one or two red flags that indicated they were not fully compliant with the study procedures or might have provided fake data. Additionally, several participants completed park surveys for parks outside the City of Chicago. In this case, participants were flagged as potentially false and excluded from the primary analyses, but were paid for participation.

Additionally, while some participants appeared to provide real data for many study elements, but the validity of specific responses were questionable (i.e., the GPS coordinates didn’t correspond with a given park location and picture didn’t help verify) or locations were redundant in outdoor surveys, these particular surveys were flagged for exclusion from main analyses. In all cases, surveys and participants that were flagged are excluded from primary analyses. Parks surveys that could not be matched to a park in the Chicago city proper are not included.

# **SUPPLEMENTARY RESULTS**

**Table S1. Mixed Effects Regressions Predicting Positive Affect**Model includes z-scored simulated weather variables. Estimates are β values with 95% CIs. Fixed effects Pseudo-*R^2^* reports the variance explained across participants, whereas total effects Pseudo-*R^2^* account for fixed effects + participant-level variability.

|  | Model 1 | Model 2 |
| --- | --- | --- |
| Intercept | 4.16 *** | 4.16 *** |
|  | [4.08, 4.25] | [4.08, 4.25] |
| Thermal Comfort | 0.14 *** | 0.14 *** |
|  | [0.10, 0.18] | [0.10, 0.19] |
| Temperature Perception |  | 0.01 |
|  |  | [-0.03, 0.05] |
| Temperature |  | 0.00 |
|  |  | [-0.05, 0.06] |
| Relative Humidity |  | 0.01 |
|  |  | [-0.04, 0.06] |
| Solar Radiation |  | 0.04 |
|  |  | [-0.01, 0.08] |
| Wind Speed |  | 0.00 |
|  |  | [-0.04, 0.04] |
| Air Pressure |  | 0.05 |
|  |  | [-0.00, 0.10] |
| N | 1946 | 1946 |
| N (UniqueID) | 313 | 313 |
| AIC | 4892.74 | 4930.19 |
| BIC | 4915.03 | 4985.93 |
| Pseudo-R^2^ (fixed) | 0.02 | 0.02 |
| Pseudo-R^2^ (total) | 0.48 | 0.47 |
| *** p < 0.001; ** p < 0.01; * p < 0.05 | | |

**Table S2. Mixed Effects Regressions Predicting Negative Affect from Comfort & Age**Estimates are β values with 95% CIs. Fixed effects Pseudo-*R^2^* reports the variance explained across participants, whereas total effects Pseudo-*R^2^* account for fixed effects + participant-level variability.

|  | Model 1 |
| --- | --- |
| Intercept | 2.09 *** |
|  | [2.02, 2.17] |
| Thermal Comfort | -0.10 *** |
|  | [-0.14, -0.07] |
| Age | -0.18 *** |
|  | [-0.25, -0.10] |
| Thermal Comfort*Age | 0.04 * |
|  | [0.00, 0.07] |
| N | 1946 |
| N (UniqueID) | 313 |
| AIC | 4460.17 |
| BIC | 4493.61 |
| Pseudo-R^2^ (fixed) | 0.05 |
| Pseudo-R^2^ (total) | 0.49 |
| *** p < 0.001; ** p < 0.01; * p < 0.05. | |

**Table S3. Mixed Effects Regressions Predicting Negative Affect (DFAW-Neg) from Temperature Perception & Age**Estimates are β values with 95% CIs. Fixed effects Pseudo-*R^2^* reports the variance explained across participants, whereas total effects Pseudo-*R^2^* account for fixed effects + participant-level variability.

|  | Model 1 |
| --- | --- |
| Intercept | 2.09 *** |
|  | [2.02, 2.17] |
| Temperature Perception | 0.04 * |
|  | [0.00, 0.07] |
| Age | -0.18 *** |
|  | [-0.25, -0.10] |
| Temperature Perception*Age | 0.00 |
|  | [-0.03, 0.03] |
| N | 1948 |
| N (UniqueID) | 313 |
| AIC | 4495.22 |
| BIC | 4528.67 |
| Pseudo-R^2^ (fixed) | 0.04 |
| Pseudo-R^2^ (total) | 0.49 |
| *** p < 0.001; ** p < 0.01; * p < 0.05. | |

**Table S4. Mixed Effects Regressions Predicting Negative Affect from Modeled Temperature & Age**Estimates are β values with 95% CIs. Fixed effects Pseudo-*R^2^* reports the variance explained across participants, whereas total effects Pseudo-*R^2^* account for fixed effects + participant-level variability.

|  | Model 1 |
| --- | --- |
| Intercept | 2.09 *** |
|  | [2.01, 2.17] |
| Temperature | -0.01 |
|  | [-0.05, 0.03] |
| Age | -0.18 *** |
|  | [-0.25, -0.10] |
| Temperature*Age | 0.02 |
|  | [-0.01, 0.06] |
| N | 1948 |
| N (UniqueID) | 313 |
| AIC | 4497.36 |
| BIC | 4530.81 |
| Pseudo-R^2^ (fixed) | 0.04 |
| Pseudo-R^2^ (total) | 0.49 |
| *** p < 0.001; ** p < 0.01; * p < 0.05. | |

**Table S5. Mixed Effects Regressions Predicting Predicting 1/x Transformed Negative Affect from Modeled Temperature & Age**Estimates are β values with 95% CIs. Fixed effects Pseudo-*R^2^* reports the variance explained across participants, whereas total effects Pseudo-*R^2^* account for fixed effects + participant-level variability.

|  | Model 1 |
| --- | --- |
| Intercept | 0.58 *** |
|  | [0.56, 0.60] |
| Temperature | 0.00 |
|  | [-0.01, 0.01] |
| Age | 0.05 *** |
|  | [0.03, 0.08] |
| Temperature*Age | -0.01 * |
|  | [-0.02, -0.00] |
| N | 1948 |
| N (UniqueID) | 313 |
| AIC | -698.61 |
| BIC | -665.16 |
| Pseudo-R^2^ (fixed) | 0.05 |
| Pseudo-R^2^ (total) | 0.51 |
| *** p < 0.001; ** p < 0.01; * p < 0.05. | |

***Acclimatization Effects: Perception Cutoff (Main Analysis)***

**Table S6. Mixed Effects Regressions Predicting Comfort from Wave and Modeled Temperature (Perception Cutoff)**

Estimates are β values with 95% CIs. Fixed effects Pseudo-*R^2^* reports the variance explained across participants, whereas total effects Pseudo-*R^2^* account for fixed effects + participant-level variability.

|  | Model 1 |
| --- | --- |
| Intercept | 4.82 *** |
|  | [4.57, 5.06] |
| Modeled Temperature | -0.53 *** |
|  | [-0.71, -0.35] |
| Wave | 0.03 |
|  | [-0.02, 0.09] |
| Modeled Temp * Wave | 0.07 *** |
|  | [0.04, 0.11] |
| N | 1946 |
| N (UniqueID) | 313 |
| AIC | 7027.06 |
| BIC | 7060.51 |
| Pseudo-R^2^ (fixed) | 0.03 |
| Pseudo-R^2^ (total) | 0.20 |
| *** p < 0.001; ** p < 0.01; * p < 0.05. | |

**Figure S1. Prediction Plot from Regression: Comfort Predicted by Modeled Temperature (T_sim) and Wave (Perception Cutoff)**


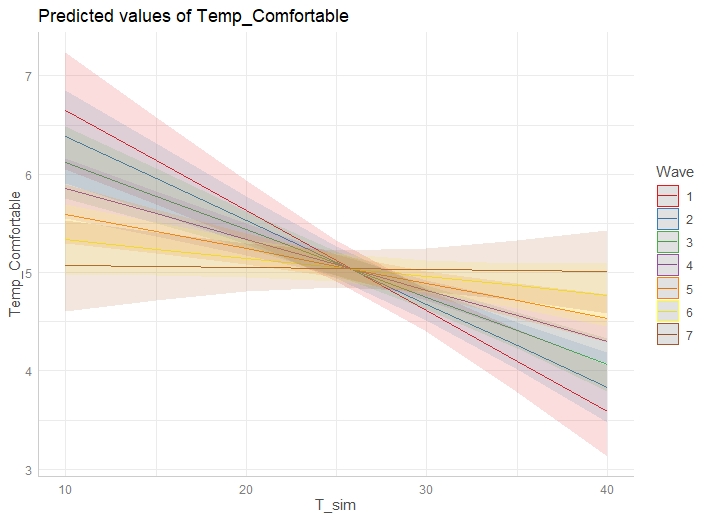


**Table S7. Mixed Effects Regressions Predicting Perception from Wave and Modeled Temperature (Perception Cutoff)**

Estimates are β values with 95% CIs. Fixed effects Pseudo-*R^2^* reports the variance explained across participants, whereas total effects Pseudo-*R^2^* account for fixed effects + participant-level variability.

|  | Model 1 |
| --- | --- |
| Intercept | 5.08 *** |
|  | [4.94, 5.21] |
| Modeled Temperature | 0.40 *** |
|  | [0.30, 0.51] |
| Wave | -0.05 ** |
|  | [-0.08, -0.02] |
| Modeled Temp * Wave | -0.02 * |
|  | [-0.05, -0.00] |
| N | 1948 |
| N (UniqueID) | 313 |
| AIC | 4928.98 |
| BIC | 4962.43 |
| Pseudo-R^2^ (fixed) | 0.11 |
| Pseudo-R^2^ (total) | 0.25 |
| *** p < 0.001; ** p < 0.01; * p < 0.05. | |

**Figure S2. Prediction Plot from Regression: Perception Predicted by Modeled Temperature (T_sim) and Wave (Perception Cutoff)**


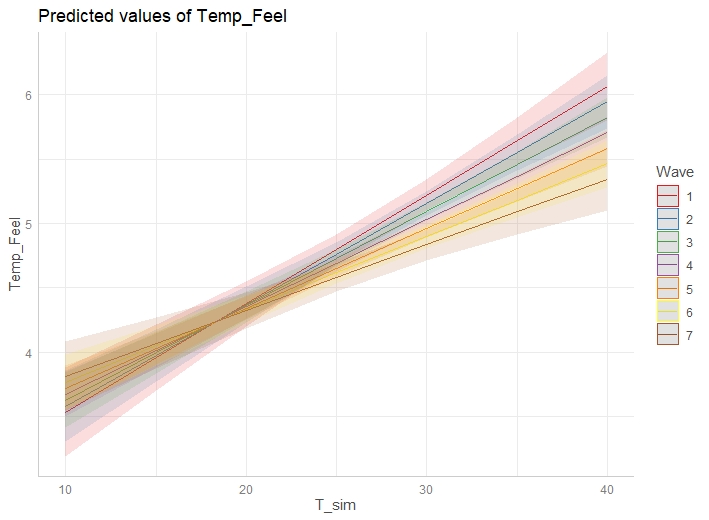


***Acclimatization Effects: Modeled Temperature > 22 C Cutoff (Secondary Analysis)***

**Table S8. Mixed Effects Regressions Predicting Comfort from Wave and Modeled Temperature (Modeled Temperature Cutoff)**

Estimates are β values with 95% CIs. Fixed effects Pseudo-*R^2^* reports the variance explained across participants, whereas total effects Pseudo-*R^2^* account for fixed effects + participant-level variability.

|  | Model 1 |
| --- | --- |
| Intercept | 4.73 *** |
|  | [4.47, 4.98] |
| Modeled Temperature | -0.35 *** |
|  | [-0.54, -0.17] |
| Wave | 0.04 |
|  | [-0.01, 0.10] |
| Modeled Temp * Wave | 0.03 |
|  | [-0.01, 0.08] |
| N | 1953 |
| N (UniqueID) | 304 |
| AIC | 7085.94 |
| BIC | 7119.40 |
| Pseudo-R^2^ (fixed) | 0.02 |
| Pseudo-R^2^ (total) | 0.21 |
| *** p < 0.001; ** p < 0.01; * p < 0.05. | |

**Figure S3. Prediction Plot from Regression: Comfort Predicted by Modeled Temperature (T_sim) and Wave (Modeled Temperature Cutoff)**

**
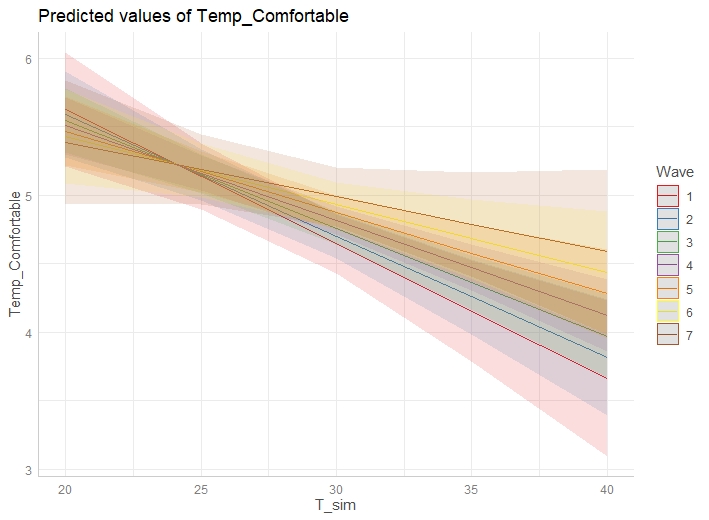
**

**Table S9. Mixed Effects Regressions Predicting Perception from Wave and Modeled Temperature (Modeled Temperature Cutoff)**

Estimates are β values with 95% CIs. Fixed effects Pseudo-*R^2^* reports the variance explained across participants, whereas total effects Pseudo-*R^2^* account for fixed effects + participant-level variability.

|  | Model 1 |
| --- | --- |
| Intercept | 5.00 *** |
|  | [4.82, 5.19] |
| Modeled Temperature | 0.37 *** |
|  | [0.24, 0.50] |
| Wave | -0.07 ** |
|  | [-0.11, -0.03] |
| Modeled Temp * Wave | -0.03 * |
|  | [-0.07, -0.00] |
| N | 1955 |
| N (UniqueID) | 304 |
| AIC | 5695.69 |
| BIC | 5729.16 |
| Pseudo-R^2^ (fixed) | 0.06 |
| Pseudo-R^2^ (total) | 0.26 |
| *** p < 0.001; ** p < 0.01; * p < 0.05. | |

**Figure S4. Prediction Plot from Regression: Perception Predicted by Modeled Temperature (T_sim) and Wave (Modeled Temperature Cutoff)**

**
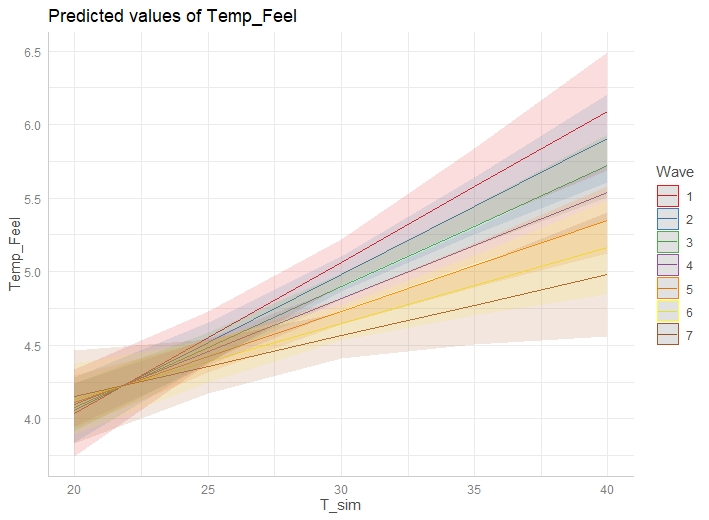
**

**References for Full Methods**

[Aron, E. N., & Aron, A. (1997). Sensory-processing sensitivity and its relation to introversion and emotionality. *Journal of Personality and Social Psychology*, *73*(2), 345–368. https://doi.org/](http://paperpile.com/b/D6CCNS/KMdS)[10.1037//0022-3514.73.2.345](http://dx.doi.org/10.1037//0022-3514.73.2.345)

[Berto, R. (2005). Exposure to restorative environments helps restore attentional capacity. *Journal of Environmental Psychology*, *25*(3), 249–259. https://doi.org/](http://paperpile.com/b/D6CCNS/iOA3)[10.1016/j.jenvp.2005.07.001](http://dx.doi.org/10.1016/j.jenvp.2005.07.001)

[Brim, O. G., Baltes, P. B., Bumpass, L. L., Cleary, P. D., Featherman, D. L., Hazzard, W. R., Kessler, R. C., Lachman, M. E., Markus, H. R., Marmot, M. G., Rossi, A. S., Ryff, C. D., & Shweder, R. A. (1999). *Midlife in the United States (MIDUS 1), 1995-1996* [Data set]. ICPSR - Interuniversity Consortium for Political and Social Research. https://doi.org/](http://paperpile.com/b/D6CCNS/RK2Q)[10.3886/ICPSR02760.V19](http://dx.doi.org/10.3886/ICPSR02760.V19)

[Buss, A. H., & Perry, M. (1992). The aggression questionnaire. *Journal of Personality and Social Psychology*, *63*(3), 452–459.](http://paperpile.com/b/D6CCNS/R9KD) <https://www.ncbi.nlm.nih.gov/pubmed/1403624>

[Fuller, R. A., Irvine, K. N., Devine-Wright, P., Warren, P. H., & Gaston, K. J. (2007). Psychological benefits of greenspace increase with biodiversity. *Biology Letters*, *3*(4), 390–394. https://doi.org/](http://paperpile.com/b/D6CCNS/5K7Q)[10.1098/rsbl.2007.0149](http://dx.doi.org/10.1098/rsbl.2007.0149)

[Hayden, M. H., Brenkert-Smith, H., & Wilhelmi, O. V. (2011). Differential Adaptive Capacity to Extreme Heat: A Phoenix, Arizona, Case Study. *Weather, Climate, and Society*, *3*(4), 269–280. https://doi.org/](http://paperpile.com/b/D6CCNS/lYfn)[10.1175/WCAS-D-11-00010.1](http://dx.doi.org/10.1175/WCAS-D-11-00010.1)

[Kirby, K. N., Petry, N. M., & Bickel, W. K. (1999). Heroin addicts have higher discount rates for delayed rewards than non-drug-using controls. *Journal of Experimental Psychology. General*, *128*(1), 78–87. https://doi.org/](http://paperpile.com/b/D6CCNS/wozW)[10.1037//0096-3445.128.1.78](http://dx.doi.org/10.1037//0096-3445.128.1.78)

[Kobau, R., Sniezek, J., Zack, M. M., Lucas, R. E., & Burns, A. (2010). Well-being assessment: An evaluation of well-being scales for public health and population estimates of well-being among US adults. *Applied Psychology. Health and Well-Being*, *2*(3), 272–297. https://doi.org/](http://paperpile.com/b/D6CCNS/sbg6)[10.1111/j.1758-0854.2010.01035.x](http://dx.doi.org/10.1111/j.1758-0854.2010.01035.x)

[Kroenke, K., & Spitzer, R. L. (2002). The PHQ-9: A new depression diagnostic and severity measure. *Psychiatric Annals*, *32*(9), 509–515. https://doi.org/](http://paperpile.com/b/D6CCNS/i2JF)[10.3928/0048-5713-20020901-06](http://dx.doi.org/10.3928/0048-5713-20020901-06)

[Malone, G. P., Pillow, D. R., & Osman, A. (2012). The General Belongingness Scale (GBS): Assessing achieved belongingness. *Personality and Individual Differences*, *52*(3), 311–316. https://doi.org/](http://paperpile.com/b/D6CCNS/klem)[10.1016/j.paid.2011.10.027](http://dx.doi.org/10.1016/j.paid.2011.10.027)

[Meidenbauer, K. L., Choe, K. W., Bakkour, A., Inzlicht, M., & Berman, M. (2021). *Characterizing the role of impulsivity in costly, reactive aggression using a novel paradigm*. https://doi.org/](http://paperpile.com/b/D6CCNS/lLJN)[10.31234/osf.io/kw3by](http://dx.doi.org/10.31234/osf.io/kw3by)

[Passmore, H.-A., & Holder, M. D. (2017). Noticing nature: Individual and social benefits of a two-week intervention. *The Journal of Positive Psychology*, *12*(6), 537–546. https://doi.org/](http://paperpile.com/b/D6CCNS/wqzx)[10.1080/17439760.2016.1221126](http://dx.doi.org/10.1080/17439760.2016.1221126)

[Patton, J. H., Stanford, M. S., & Barratt, E. S. (1995). Factor structure of the Barratt impulsiveness scale. *Journal of Clinical Psychology*, *51*(6), 768–774. https://doi.org/](http://paperpile.com/b/D6CCNS/2F46)[10.1002/1097-4679(199511)51:6<768::aid-jclp2270510607>3.0.co;2-1](http://dx.doi.org/10.1002/1097-4679(199511)51:6%3C768::aid-jclp2270510607%3E3.0.co;2-1)

[Russell, E., & Daniels, K. (2018). Measuring affective well-being at work using short-form scales: Implications for affective structures and participant instructions. *Human Relations; Studies towards the Integration of the Social Sciences*, *71*(11), 1478–1507. https://doi.org/](http://paperpile.com/b/D6CCNS/8LTQ)[10.1177/0018726717751034](http://dx.doi.org/10.1177/0018726717751034)

[Ryan, R. M., Weinstein, N., Bernstein, J., Brown, K. W., Mistretta, L., & Gagné, M. (2010). Vitalizing effects of being outdoors and in nature. *Journal of Environmental Psychology*, *30*(2), 159–168. https://doi.org/](http://paperpile.com/b/D6CCNS/PbjO)[10.1016/j.jenvp.2009.10.009](http://dx.doi.org/10.1016/j.jenvp.2009.10.009)

[Schertz, K. E., Bowman, J. E., Kotabe, H., Layden, E. A., Zhen, J., Lakhtakia, T., Lyu, M., Paraschos, O., Van Hedger, S. C., Vohs, K., & al., E. (2022). *Nature’s path to thinking about others and the surrounding environment*. https://doi.org/](http://paperpile.com/b/D6CCNS/YZkL)[10.31234/osf.io/q27bc](http://dx.doi.org/10.31234/osf.io/q27bc)

[Schertz, K. E., Bowman, J. E., Kotabe, H. P., Layden, E. A., Zhen, J., Lakhtakia, T., Lyu, M., Paraschos, O. A., Van Hedger, S. C., Rim, N. W., Vohs, K. D., & Berman, M. G. (2022). Environmental influences on affect and cognition: A study of natural and commercial semi-public spaces. *Journal of Environmental Psychology*, *83*, 101852. https://doi.org/](http://paperpile.com/b/D6CCNS/xny3)[10.1016/j.jenvp.2022.101852](http://dx.doi.org/10.1016/j.jenvp.2022.101852)

[Schultz, P. W. (2002). Inclusion with Nature: The Psychology Of Human-Nature Relations. In P. Schmuck & W. P. Schultz (Eds.), *Psychology of Sustainable Development* (pp. 61–78). Springer US. https://doi.org/](http://paperpile.com/b/D6CCNS/m3CP)[10.1007/978-1-4615-0995-0_4](http://dx.doi.org/10.1007/978-1-4615-0995-0_4)

[Soto, C. J., & John, O. P. (2017). Short and extra-short forms of the Big Five Inventory–2: The BFI-2-S and BFI-2-XS. *Journal of Research in Personality*, *68*, 69–81. https://doi.org/](http://paperpile.com/b/D6CCNS/jnXL)[10.1016/j.jrp.2017.02.004](http://dx.doi.org/10.1016/j.jrp.2017.02.004)

[Trapnell, P. D., & Campbell, J. D. (1999). Private self-consciousness and the five-factor model of personality: Distinguishing rumination from reflection. *Journal of Personality and Social Psychology*, *76*(2), 284–304. https://doi.org/](http://paperpile.com/b/D6CCNS/uT13)[10.1037/0022-3514.76.2.284](http://dx.doi.org/10.1037/0022-3514.76.2.284)

[Ulrich, R. S., Simons, R. F., Losito, B. D., Fiorito, E., Miles, M. A., & Zelson, M. (1991). Stress recovery during exposure to natural and urban environments. *Journal of Environmental Psychology*, *11*(3), 201–230. https://doi.org/](http://paperpile.com/b/D6CCNS/niSl)[10.1016/S0272-4944(05)80184-7](http://dx.doi.org/10.1016/S0272-4944(05)80184-7)
